# A novel nomogram to stratify quality of life among advanced cancer patients with spinal metastatic disease after examining demographics, dietary habits, therapeutic interventions, and mental health status

**DOI:** 10.1186/s12885-022-10294-z

**Published:** 2022-11-23

**Authors:** Yue Li, Ze Long, Xiuju Wang, Mingxing Lei, Chunzi Liu, Xiaolin Shi, Yaosheng Liu

**Affiliations:** 1 Department of Oncology, The Fifth Medical Center of Chinese PLA General Hospital, No. 8, Fengtaidongda Rd, Beijing, 100071 People’s Republic of China; 2 Department of Orthopedics, The Second Xiangya Hospital of Central South University, Changsha, China; 3 Department of Orthopedic Surgery, Hainan Hospital of Chinese PLA General Hospital, Sanya, China; 4 Chinese PLA Medical School, Beijing, China; 5 Department of Orthopedic Surgery, the Fourth Medical Center of Chinese PLA General Hospital, No. 8, Fengtaidongda Rd, Beijing, 100071 People’s Republic of China; 6 Department of Orthopedic Surgery, The Second Affiliated Hospital of Zhejiang Chinese Medical University, No. 318, Chaowang Road, Hangzhou, 310053 People’s Republic of China; 7 National Clinical Research Center for Orthopedics, Sports Medicine & Rehabilitation, Chinese PLA General Hospital, Beijing, China

**Keywords:** Spine metastases, Quality of life, Nomogram, Risk factors, Mental health

## Abstract

**Background:**

It would be very helpful to stratify patients and direct patient selection if risk factors for quality of life were identified in a particular population. Nonetheless, it is still challenging to forecast the health-related quality of life among individuals with spinal metastases. The goal of this study was to stratify patient’s populations for whom the assessment of quality of life should be encouraged by developing and validating a nomogram to predict the quality of life among advanced cancer patients with spine metastases.

**Methods:**

This study prospectively analyzed 208 advanced cancer patients with spine metastases, and collected their general characteristics, food preferences, addictions, comorbidities, therapeutic strategies, and mental health status. The functional assessment of cancer therapy-general (FACT-G) and hospital anxiety and depression scale (HADS) were used to assess quality of life and mental health, respectively. The complete cohort of patients was randomly divided into two groups: a training set and a validation set. Patients from the training set were conducted to train and develop a nomogram, while patients in the validation set were performed to internally validate the nomogram. The nomogram contained significant variables discovered using the least absolute shrinkage and selection operator (LASSO) approach in conjunction with 10-fold cross-validation. The nomogram’s predictive ability was assessed utilizing discrimination, calibration, and clinical usefulness. Internal validation was also completed using the bootstrap method after applying 500 iterations of procedures. A web calculator was also developed to promote clinical practice.

**Results:**

Advance cancer patients with spinal metastases had an extremely low quality of life, as indicated by the average FACT-G score of just 60.32 ± 20.41. According to the LASSO and 10-fold cross-validation, Eastern Cooperative Oncology Group (ECOG) score, having an uncompleted life goal, preference for eating vegetables, chemotherapy, anxiety status, and depression status were selected as nomogram predictors. In the training set, the area under the receiver operating characteristic curve (AUROC) was 0.90 (95% CI: 0.84–0.96), while in the validation set, it was 0.85 (95% CI: 0.78–0.93). They were 0.50 (95% CI: 0.41–0.58) and 0.44 (95% CI: 0.33–0.56), respectively, for the discrimination slopes. The nomogram had favorable capacity to calibrate and was clinically useful, according to the calibration curve and decision curve analysis. When compared to patients in the low-risk group, patients in the high-risk group were above four times more likely to experience a poor quality of life (82.18% vs. 21.50%, *P* < 0.001). In comparison to patients in the low-risk group, patients in the high-risk group also exhibited significant higher levels of anxiety and depression. The webpage for the web calculator was https://starshiny.shinyapps.io/DynNomapp-lys/.

**Conclusions:**

This study suggests a nomogram that can be applied as a practical clinical tool to forecast and categorize the quality of life among patients with spine metastases. Additionally, patients with poor quality of life experience more severe anxiety and depression. Effective interventions should be carried out as soon as possible, especially for patients in the high-risk group, to improve their quality of life and mental health condition.

**Supplementary Information:**

The online version contains supplementary material available at 10.1186/s12885-022-10294-z.

## Background

Cancer is a severe global public health issue. Recent global cancer statistics estimated that 19.3 million new cancer cases were diagnosed and almost 10.0 million cancer deaths occurred in 2020 alone [1]. Even worse, the burden of cancer is projected to rise to 28.4 million cases globally by 2040, a 47% increase from 2020, and the increase could be up to 64 to 95% in transitioning countries because of demographic changes, exacerbated globalization and economy [1].

Spine metastases are a severe consequence of cancer patients, and the incidence of spine metastases has significantly increased due to growing cancer patients and prolonged life expectancy among those patients [2]. This disease is featured by intractable severe back pain, neurological sequelae, and even incontinence and disability, all of which could have a significant impact on the quality of life among patients with a limited life expectancy [3]. Thus, maintaining or promoting the patient’s quality of life to the greatest extent possible is the primary objective of contemporary treatment for spinal metastases.

However, even though studies have pointed out that the therapeutic options, like radiotherapies and spinal procedures, could improve the quality of life for patients with spine metastases [2], it was difficult to predict the quality of life in advance, and inappropriate patient’s selection and interventions could even cause harms to patients and deteriorate their quality of remaining life. Fortunately, a number of factors including age [4], gender [4], sarcopenia [5], financial difficulty scores [6], Eastern Cooperative Oncology Group (ECOG) scores, the percentage weight loss, and modified Glasgow Prognostic score [7], have been found to be associated with quality of life. The majority of the aforementioned characteristics, nonetheless, were studied in general cancer patients, and there is very little information on risk factors linked to low quality of life, particularly in the cases of spine metastases. Recent studies suggested that neurologic impairments [8] and surgery [9] may have an impact on the quality of life especially among patients with spine metastases. It would be very helpful to stratify patients and direct patient selection if risk factor for quality of life were identified in a particular demographic. Quality of life should also be easily accessed to support shared clinical decision-making for clinicians. Additionally, clinicians would benefit greatly from a prediction model to assess the quality of life since effective interventions would be possible for patients.

The study’s goal was to create a nomogram to categorize quality of life in advanced cancer patients with spine metastases, and to further internally validate the nomogram’s efficacy in making predictions.

## Methods

### Sample population and study design

Between April 2021 and April 2022, 208 advanced cancer patients with spine metastases admitted at the Fifth Medical Center of Chinese PLA General Hospital were prospectively examined. The study collected patient’s general characteristics, food preferences, comorbidities, therapeutic strategies, mental health status, and quality of life, and data from their medical records were cross-checked. Contradictory data were discussed and confirmed with at least two doctors. Patient’s mental health and quality of life were evaluated using questionnaires. When patients participated in the interview, doctors were always available to them as a further assurance of the validity of the data. Patients with spine metastases which were confirmed by tissue biopsy and radiography such as magnetic resonance imaging (MRI) and/or computer tomography (CT) were included in the study. Patients were excluded, if they (1) were younger than 18 years of age, (2) were reluctant to participant in the study, (3) had been diagnosed with psychiatric disorders; (4) had metastases to the extremities or rib rather than the spine; (5) had an expected survival of less than 3 months according to Tomita [10], Takahashi [11], and Lei and Liu [12] scores [13]; (6) had a ECOG score of 5.

The complete cohort of patients was randomly divided into two groups: a training set and a validation set. Patients from the training set were conducted to propose the nomogram, while patients from the validation set were performed to internally validation the nomogram. This study was approved by the Ethics Committee of the Fourth Medical Center of Chinese PLA General Hospital. Informed written consent was obtained from all patients, and all data were analyzed anonymously. This study abided by the Helsinki Declaration.

### Outcome: quality of life

The functional assessment of cancer therapy-general (FACT-G) [14] was used to assess quality of life. The FACT-G was widely utilized and verified to assess the quality of life among cancer patients. This is a self-reported tool with 27 items and four subscales. The four subscales include the (1) physical well-being score (7 items), (2) social well-being score (7 items), (3) emotional well-being score (6 items), and functional well-being score (7 items). Each item receives a rating on a 0–4 Likert-type scale. The overall FACT-G scores, which ranged from 0 to 108, were the sum of the four subscales, and total scores of each subscale were derived from the sum of each item in each subscale. A higher FACT-G score indicates better quality of life. Based on previous studies, the FACT-G score peaked at 3 months, and were largely maintained throughout the follow-up period among patients with spine metastasis [15]. Therefore, 3 months after being discharged from hospital, patients were asked to self-report how they actual felt over the previous 2 weeks as part of the FACT-G score collection. In the study, to further improve the specificity of suffering from poor quality of life among spine metastases patients, we defined that a FACT-G score of less than 60, which was the median of FACT-G scores among the entire patients in the study, was classified as relatively poor quality of life.

### Collection of risk factors and descriptions

The 25 risk factors that were collected and recorded in this study comprised their general characteristics, food preferences, addictions, comorbidities, therapeutic strategies, and mental health status. These risk factors may potentially relate to the low level of quality of life. Patient’s general characteristics included age, sex, nationality, marital status, education level, caregivers, and having an uncompleted life goal. Life style and health behavior mainly referred to patient’s food preferences including preference for eating vegetables and eating roasted food, and patient’s addictions including smoking and drinking status. Patient’s cancer-related information included primary cancer type, visceral metastasis, time since knowing cancer diagnosis, and eastern cooperative oncology group (ECOG) scores. Patient’s comorbidities included hypertension, diabetes, and coronary heart disease. Patient’s therapeutic strategies included surgery for primary cancer site, surgery for spine metastases, radiotherapy, chemotherapy, and economic burden due to cancer treatments. The hospital anxiety and depression Scale (HADS) was used to assess mental health, including anxiety and depression [16, 17]. HADS is a useful and validated instrument for patients during hospitalization and hospital outpatients. It consists of two subscales, one of which measures anxiety and the other of which measures depression. Each subscale includes 7 items, and patients were asked to report how they felt on a four-point (0–3) score over the last 2 weeks. Scores for anxiety and depression vary from 0 to 21 respectively: a score of 0 to 7 indicates absence of anxiety or depression, 8 to 10 indicates skeptical anxiety or depression, and 11 or above means presence of an anxiety or depression disorder. The aforementioned data was collected and verified in the medical records or through self-reporting. The item “having an uncompleted life goal” was self-reported by participants according to their actual status. In the study, open surgery for patients with spine metastases mainly consisted of open pedicular screw fixation with or without excision of tumor via a resection of vertebral body or laminectomy, whereas minimally invasive surgery for patients with spine metastases mainly included percutaneous vertebroplasty and balloon kyphoplasty. Open surgery for primary cancer mainly included thoracotomy for thoracic cancers and laparotomy for visceral malignant tumors, while minimally invasive surgery for primary cancer typically involved laparoscopic and thoracoscopic surgery.

### Nomogram establishment

The nomogram contained significant variables determined by the least absolute shrinkage and selection operator (LASSO) combined with ten-fold cross-validation. Subgroup analysis was conducted among selected variables. The LASSO method, a penalized regression model, is capable of selecting variables and discarding confounding variables by minimizing the comparatively irrelevant variables’ coefficient to 0. The nomogram did not include variables with a coefficient of 0. The ten-fold cross-validation approach was also used to adjust the parameter λ so that relevant variables could be accurately identified. After relevant variables were identified, and the study used the multiply logistic regression model [18] to train and develop the prediction model using the R package called “rms”. The targets of this training were to construct the prediction nomogram based on the above LASSO-selected variables. Next, the prediction model was presented as the format of nomogram with the R package of “regplot”. In order to encourage clinical application, a web-based calculator was developed in “shinyapps” using the R package of “DynNom”.

### Nomogram validation

The discriminative capacity of the nomogram was evaluated using the area under the receiver operating characteristic curve (AUROC). A perfect fit is shown by an AUROC of 1, whereas a random chance is indicated by a value of 0.5 [19]. Generally, an AUROC of more than 0.7 suggests a useful estimate. Discrimination slope was also used to evaluate the nomogram [20], and it was the mean difference of predicted risk probability between patients with and without a positive event (relatively poor quality of life).

Calibration curve was plotted using the bootstrap method after applying 500 iterations of procedures, in order to assess the calibration ability of the nomogram. Decision curve analysis was performed to assess the clinical benefits and utility of the nomogram. Decision curve analysis is widely used to evaluate the clinical benefit of models [21], and it is plotted with different threshold probabilities against net benefits. The two references in the decision curve were treat-for-all and treat-for-none schedule. The former schedule indicates the highest clinical costs, and the latter schedule indicates no clinical benefit. In addition, the study calculated accuracy, sensitivity, specificity, recall index, and Youden index for the nomogram.

The overall prediction performance was evaluated using Brier score and Brier_scaled_ score. The Brier score is a quadratic scoring rule, which is defined as the squared differences between actual binary outcomes Y (negative events vs. positive events) and predicted probability p are calculated: (Y–p)^2^ [20]. The Brier score for a prediction nomogram can range from 0, which indicates a perfect nomogram, to 1, which denotes an uninformative prediction model with a 50% likelihood of the outcome. Of note, a Brier score of more than 0.25 is considered as a worthless nomogram. The maximum Brier_scaled_ score in an uninformative model can be calculated as follows: Brier_scaled_ = 1 – Brier/Briermax, and Briermax = mean (*p*) × (1– mean (*p*)). Therefore, the range of the Brier_scaled_ score is 0 to 100.00%. A lower Brier score or Brier_scaled_ score indicates better overall performance of the nomogram.

### Statistical analysis

Continuous characteristics were described as mean ± standard deviation, while categorical characteristics were described as proportions. The difference between two groups was compared using the Chi-square test for categorical characteristics and the student’s *t* test or Wilcoxon two-sample test for continuous characteristics. The predicted risk probability between patients with poor quality of life (positive events) and patients without poor quality of life (negative events) in the training and validation sets was visualized using probability density curves. The cut-off points were used for the risk stratification among the entire patients. The four FACT-G subscales were represented using radar charts, which also revealed how the training and validation sets, negative and positive groups, and low-risk and high-risk groups were distributed. Violin plots were used to visualize the anxiety and depression scores also between the training and validation sets, the negative and positive event groups, and the low-risk and high-risk groups. The R programming language software was used to train, develop, and present the prediction model, and the R programming language software (Version 4.1.2, http://www.r-project.org/) and the SAS 9.4 software were used to carry out the statistical analyses. *P* values lower than 0.05 was considered as significant (two-tailed).

## Results

### Patient’s demographics and clinical characteristics

Analysis was performed on a cohort of 208 patients, with a mean age of 58.74 ± 12.00 years. The most patients were the Han nationality (96.63%), were married (93.27%), were cared for by a spouse (64.90%), did not smoke (57.21%), and did not consume alcohol (73.56%). Only 24.52% patients had hypertension, 9.62% patients had diabetes, and 7.69% patients had coronary heart disease, hence there were not many patients with severe comorbidities. Lung cancer was the most common cancer type with a proportion of 57.21%. However, the financial burden associated with cancer treatment was reported to be significant by 54.33% of patients. In addition, 43.27% patients had visceral metastasis, 60.58% patients treated with radiotherapy, and 60.58% patients treated with chemotherapy. Table 1 provides a summary of further information on the demographics and clinical traits of patients. The mean FACT-G score was only 60.32 ± 20.41, which indicated that, in comparison to typical populations, patients with spine metastases generally suffered from very poor quality of life. Additionally, the mental health status of those patients was far from satisfaction because up to 52.40% of patients reported having anxious symptoms and 48.45% reported having depressive symptoms. When a FACT-G score of less than 60 was deemed to indicate the poor quality of life, 50.96% of patients experienced this. A 50:50 split of patients into the training and validation sets was done at random. The distribution of all other characteristics was comparable between the two groups (*P* > 0.05, Table 1), with the exception of drinking status and hypertension. In detail, the proportion of poor quality of life was the same between the training and validation sets (Both 50.96%).

**Table 1:** Patient’s baseline clinical characteristics and comparisons between the training and validation groups

Characteristics	Patients (***n*** = 208)	Training group (***n*** = 104)	Validation group (***n*** = 104)	P
Age (mean ± SD, years)	58.74 ± 12.00	58.12 ± 12.15	59.36 ± 11.88	0.46 ^a^
Sex				0.68
Male	51.44% (107/208)	50.00% (52/104)	52.88% (55/104)	
Female	48.56% (101/208)	50.00% (52/104)	47.12% (49/104)	
Nationality				0.70
Han nationality	96.63% (201/208)	96.15% (100/104)	97.12% (101/104)	
Ethnic minorities	3.37% (7/208)	3.85% (4/104)	2.88% (3/104)	
Marital status				0.58
Married	93.27% (194/208)	92.31% (96/104)	94.23% (98/104)	
Single	6.73% (14/208)	7.69% (8/104)	5.77% (6/104)	
Education				
Primary education	35.58% (74/208)	35.58% (37/104)	35.58% (37/104)	0.69
Senior high school	35.10% (73/208)	32.69% (34/104)	37.50% (39/104)	
University or above	29.33% (61/208)	31.73% (33/104)	26.92% (28/104)	
Caregivers				0.90
Spouse	64.90% (135/208)	64.42% (67/104)	65.38% (68/104)	
Other family members	18.75% (39/208)	19.23% (20/104)	18.27% (19/104)	
Support workers	4.81% (10/208)	5.77% (6/104)	3.85% (4/104)	
No caregivers	11.54% (24/208)	10.58% (11/104)	12.50% (13/104)	
Preference for eating vegetables				0.68
No	13.46% (28/208)	14.42% (15/104)	12.50% (13/104)	
Yes	86.54% (180/208)	85.58% (89/104)	87.50% (91/104)	
Preference for eating roasted food				0.64
No	90.38% (188/208)	91.35% (95/104)	89.42% (93/104)	
Yes	9.62% (20/208)	8.65% (9/104)	10.58% (11/104)	
Smoking status				0.30
No	57.21% (119/208)	62.50% (65/104)	51.92% (54/104)	
Quitting smoking	23.56% (49/208)	21.15% (22/104)	25.96% (27/104)	
Current smoking	19.23% (40/208)	16.35% (17/104)	22.12% (23/104)	
Drinking status				0.03
No	73.56% (153/208)	76.92% (80/104)	70.19% (73/104)	
Quitting drinking	18.75% (39/208)	12.50% (13/104)	25.00% (26/104)	
Current drinking	7.69% (16/208)	10.58% (11/104)	4.81% (5/104)	
Hypertension				0.02
No	75.48% (157/208)	82.69% (86/104)	68.27% (71/104)	
Yes	24.52% (51/208)	17.31% (18/104)	31.73% (33/104)	
Diabetes				0.64
No	90.38% (188/208)	89.42% (93/104)	91.35% (95/104)	
Yes	9.62% (20/208)	10.58% (11/104)	8.65% (9/104)	
Coronary heart disease				0.30
No	92.31% (192/208)	94.23% (98/104)	90.38% (94/104)	
Yes	7.69% (16/208)	5.77% (6/104)	9.62% (10/104)	
Time since knowing cancer diagnosis				0.05
< 3 months	17.79% (37/208)	15.38% (16/104)	20.19% (21/104)	
≧3 months and < 6 months	10.10% (21/208)	7.69% (8/104)	12.50% (13/104)	
≧6 months and < 12 months	10.10% (21/208)	15.38% (16/104)	4.81% (5/104)	
≧12 months	62.02% (129/208)	61.54% (64/104)	62.50% (65/104)	
Primary cancer type				0.13
Lung cancer	57.21% (119/208)	50.96% (53/104)	63.46% (66/104)	
Liver cancer	4.81% (10/208)	3.85% (4/104)	5.77% (6/104)	
Gastrointestinal cancer	7.69% (16/208)	7.69% (8/104)	7.69% (8/104)	
Breast cancer	9.62% (20/208)	9.62% (10/104)	9.62% (10/104)	
Others	20.67% (43/208)	27.88% (29/104)	13.46% (14/104)	
Visceral metastases				1.00
No	56.73% (118/208)	56.73% (59/104)	56.73% (59/104)	
Yes	43.27% (90/208)	43.27% (45/104)	43.27% (45/104)	
Surgery for primary cancer site				0.13
Open surgery	19.71% (41/208)	25.00% (26/104)	14.42% (15/104)	
Minimally invasive surgery	20.67% (43/208)	21.15% (22/104)	20.19% (21/104)	
None	59.62% (124/208)	53.85% (56/104)	65.38% (68/104)	
Surgery for spine metastasis				0.63
Open surgery	15.87% (33/208)	18.27% (19/104)	13.46% (14/104)	
Minimally invasive surgery	54.81% (114/208)	52.88% (55/104)	56.73% (59/104)	
None	29.33% (61/208)	28.85% (30/104)	29.81% (31/104)	
Radiotherapy				0.26
No	39.42% (82/208)	35.58% (37/104)	43.27% (45/104)	
Yes	60.58% (126/208)	64.42% (67/104)	56.73% (59/104)	
Chemotherapy				0.09
No	39.42% (82/208)	33.65% (35/104)	45.19% (47/104)	
Yes	60.58% (126/208)	66.35% (69/104)	54.81% (57/104)	
Economic burden due to cancer treatments				0.83
None	2.88% (6/208)	2.88% (3/104)	2.88% (3/104)	
Mild	10.58% (22/208)	8.65% (9/104)	12.50% (13/104)	
Moderate	32.21% (67/208)	33.65% (35/104)	30.77% (32/104)	
Severe	54.33% (113/208)	54.81% (57/104)	53.85% (56/104)	
Having an uncompleted life goal				0.52
No	24.04% (50/208)	22.12% (23/104)	25.96% (27/104)	
Yes	75.96% (158/208)	77.88% (81/104)	74.04% (77/104)	
ECOG score				0.54
0	6.73% (14/208)	6.73% (7/104)	6.73% (7/104)	
1	34.13% (71/208)	35.58% (37/104)	32.69% (34/104)	
2	29.81% (62/208)	31.73% (33/104)	27.88% (29/104)	
3	11.54% (24/208)	7.69% (8/104)	15.38% (16/104)	
4	17.79% (37/208)	18.27% (19/104)	17.31% (18/104)	
Anxiety				0.69
No	47.60% (99/208)	46.15% (48/104)	49.04% (51/104)	
Skeptical	20.67% (43/208)	23.08% (24/104)	18.27% (19/104)	
Yes	31.73% (66/208)	30.77% (32/104)	32.69% (34/104)	
Depression				0.30
No	51.44% (107/208)	51.92% (54/104)	50.96% (53/104)	
Skeptical	19.23% (40/208)	15.38% (16/104)	23.08% (24/104)	
Yes	29.33% (61/208)	32.69% (34/104)	25.96% (27/104)	
FACT-G scores	60.32 ± 20.41	60.83 ± 20.18	59.82 ± 20.73	0.85 ^b^
Physical well-being	14.41 ± 7.22	14.23 ± 7.35	14.60 ± 7.11	0.70 ^b^
Social well-being	18.62 ± 5.82	18.90 ± 5.63	18.33 ± 6.02	0.51 ^b^
Emotional well-being	14.24 ± 5.7	14.20 ± 5.58	14.28 ± 5.84	0.99 ^b^
Functional well-being	13.05 ± 7.14	13.49 ± 7.48	12.62 ± 6.78	0.42 ^b^

*SD* standard deviation*, ECOG* eastern cooperative oncology group*, FACT-G* functional assessment of cancer therapy-general

^a^indicates the student’s *t* test

^b^indicates the Wilcoxon two-sample test

### Nomogram construction

**Fig. 1:**
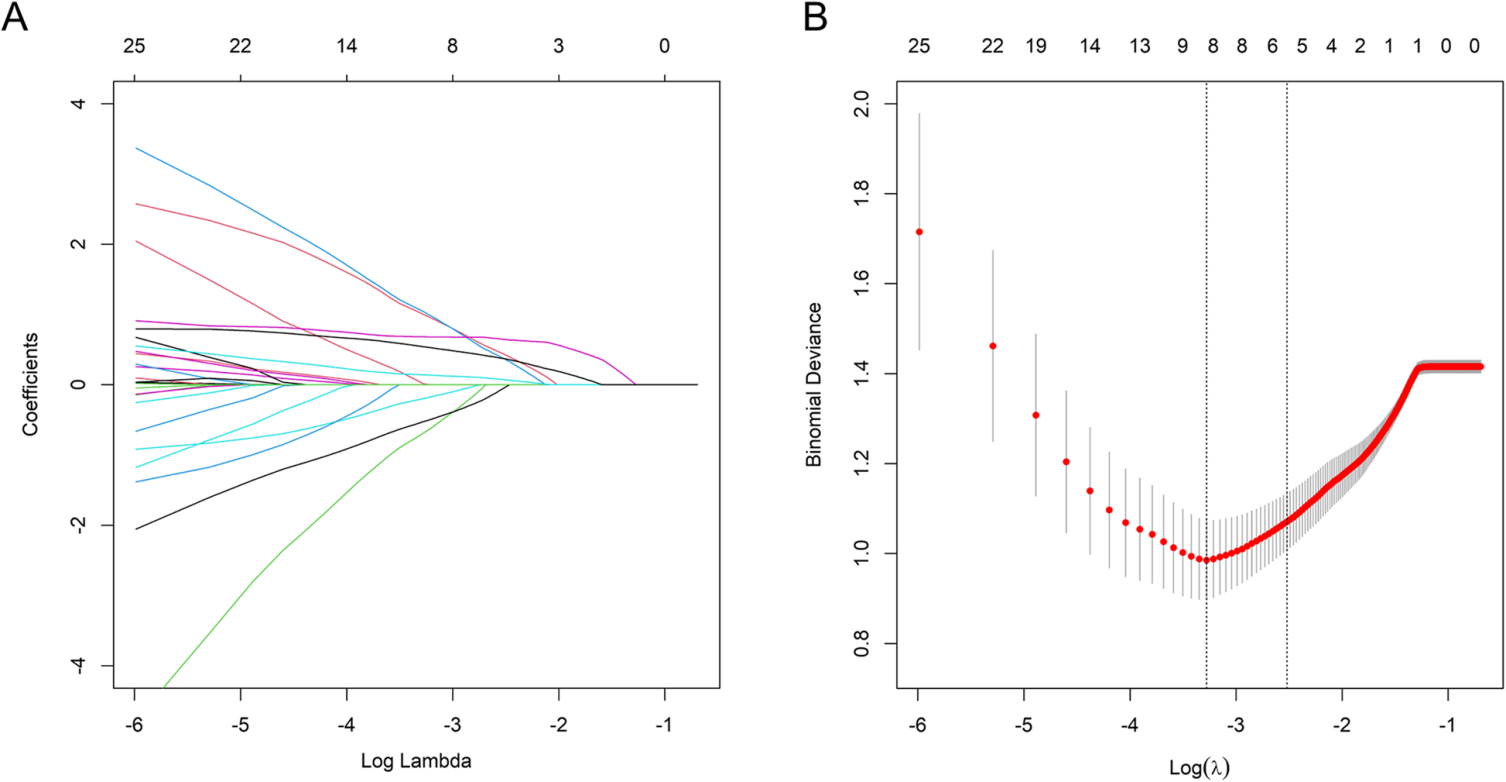
Selection of variables using the LASSO (least absolute shrinkage and selection operator) method in conjunction with ten-fold cross-validation. **A** LASSO coefficient profiles of all variables. Coefficient profiles were plotted against the log lambda sequence. **B** Selection of appropriate parameters (λ). Binomial deviance was plotted against log lambda. Dotted vertical lines were drawn at the optimal values by the minimum criteria (the left dotted vertical line) and the one standard error of the minimum criteria (the right dotted vertical line)

**Fig. 2:**
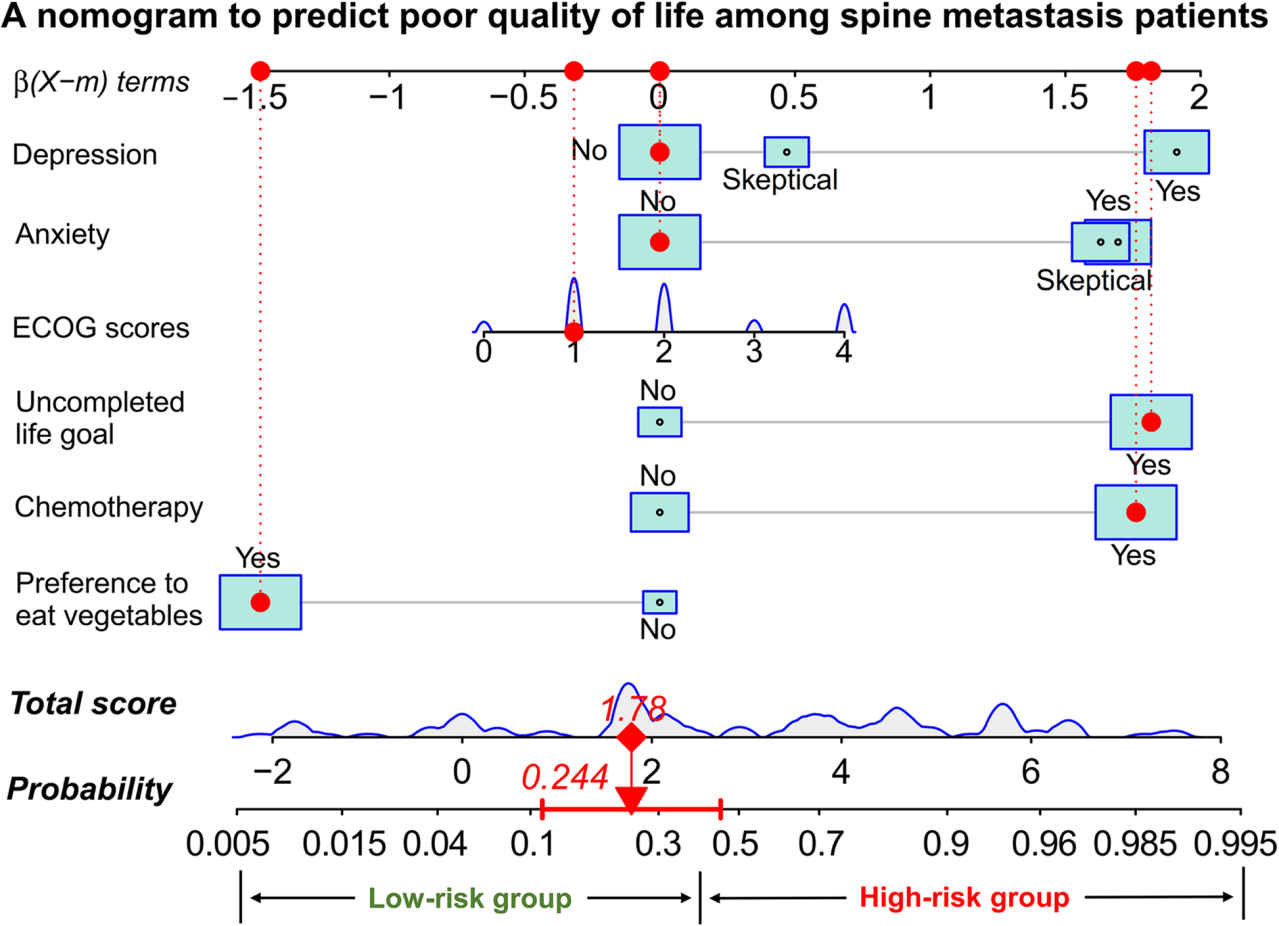
Nomogram to predict relatively poor quality of life among advanced cancer patients with spine metastases. The distributions of the total scores and ECOG are shown in a density plot. For proportion variables, their distributions are presented by the size of box, with a larger size indicating a higher proportion

In the training set, six variables, including ECOG score, having an uncompleted life goal, preference for eating vegetables, chemotherapy, anxiety status, and depression status, were significant and included in the nomogram, according to the LASSO method and ten-fold cross-validation (Fig. 1). This study developed the nomogram to predict the risk probability of poor quality of life among advanced cancer patients with spine metastases, and it demonstrated that higher levels of anxiety and depression, a higher ECOG score, having uncompleted life goals, and chemotherapy were associated with a higher risk probability of suffering from poor quality of life, whereas preference for eating vegetables was a protective factor for poor quality of life (Fig. 2).

An illustration of using the nomogram to predict the risk likelihood of poor quality of life in a specific given patient was provided in the nomogram. In this particular situation, the patients did not have any signs of anxiety and depression, had an ECOG score of 1, had an uncompleted life goal, received with chemotherapy, and preferred to eat vegetables. Each variable’s score could be determined by referring to the β (X-m) terms axis. For instance, in this scenario, anxiety and depression both received 0, and preference for eating vegetables received − 1.5 points. The total score was determined based on the sum of the six scores, and the value displayed on the total score axis was 1.78. The total score varied from − 2 to 8, and a higher score indicated that patients with spine metastases were more likely to have a poor quality of life. The probability of having poor quality of life could be determined by looking dawn to the probability axis, and in this instance, the predicted risk was 0.244, and it meant a risk of low quality of life for this patient of 24.4%.

The study further developed a web calculator to aid clinical practice, and the address of the website was https://starshiny.shinyapps.io/DynNomapp-lys/. By selecting the appropriate items and clicking the “Predict” button on the website, one can determine the predicted risk probability of the outcome. The website offered a graphical summary, numerical summary, and model summary.

### Subgroup analysis of nomogram predictors

Subgroup analyses were conducted in the study to further elucidate the above findings for the selected predictors in the nomogram. Patients treated with chemotherapy had near two-time greater odds of having poor quality of life compared to patients without chemotherapy (61.9% vs. 34.1%, *P* < 0.001). Additionally, as for the four subscales of FACT-G—physical well-being score (*P* < 0.001), social well-being score (*P* = 0.027), emotional well-being score (*P* = 0.015), and functional well-being score (*P* = 0.016)—all supported the finding that patients receiving chemotherapy had lower scores than those receiving no chemotherapy. In addition, subgroup analyses further confirmed the trend in the variables of depression, anxiety, ECOG score, having an uncompleted life goal, and preference for eating vegetables: Higher levels of anxiety and depression, a higher ECOG score, and having uncompleted life goals were associated with a higher risk probability of poor quality of life, whereas preference for eating vegetables was a protective factor for poor quality of life. To learn more about how visceral metastases might affect those patients’ quality of life, subgroup analysis was also carried out. It demonstrated that patients with visceral metastases truly had significantly a lower level of FACT-G score as compared to patients without visceral metastases (54.68 ± 18.35 vs. 64.63 ± 20.93, *P* < 0.001). Nonetheless, the variable of visceral metastases was not selected by the LASSO in the study. Patients with visceral metastasis were equally distributed between the training and validation sets (43.27% vs. 43.27%, *P* = 1.000, Table 1). Although this variable was not included in the nomogram, it was also applicable among spine metastases patients with visceral metastases.

### Nomogram validation

**Fig. 3:**
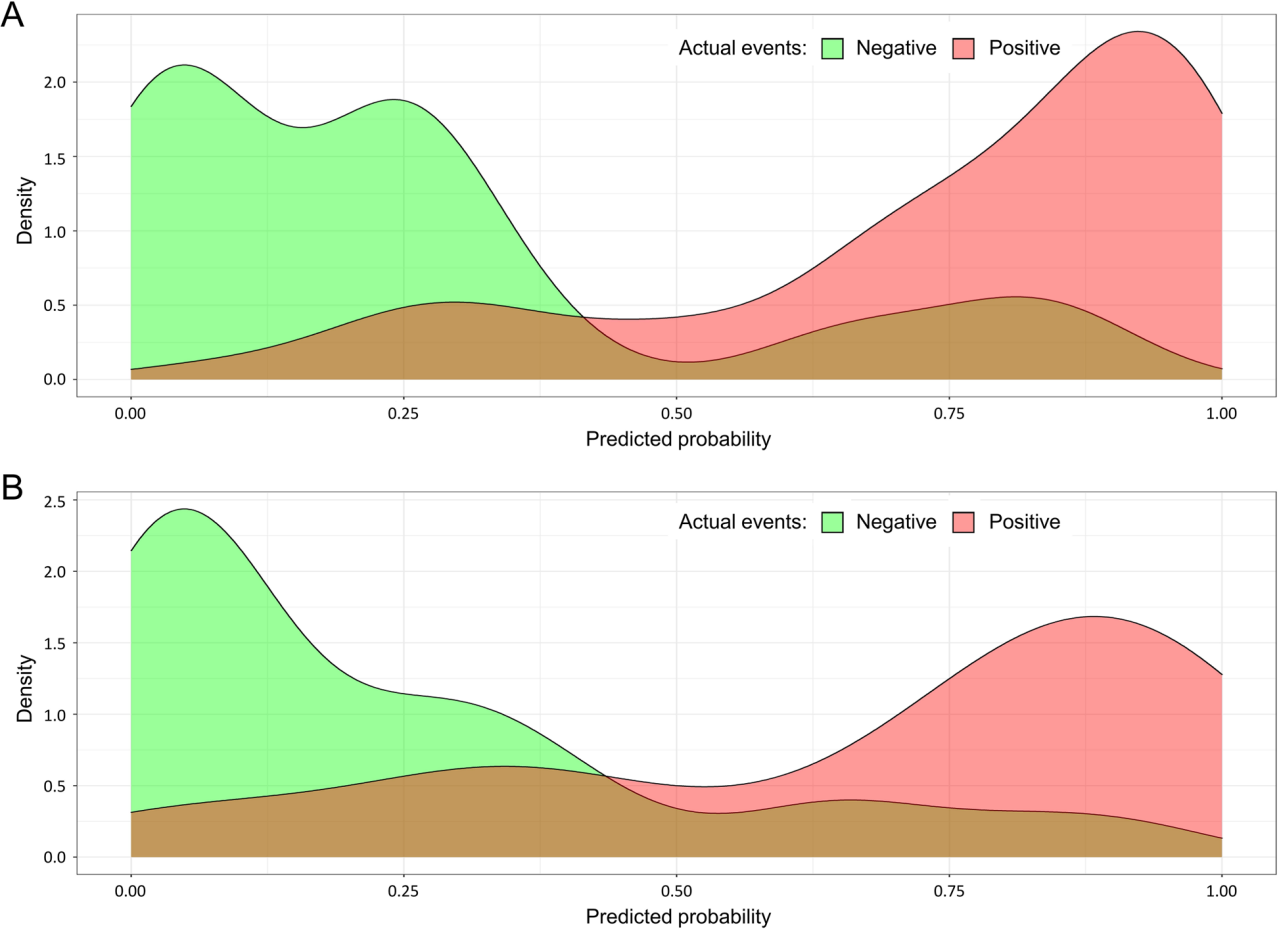
Probability density curve between patients without (negative, green) and with (positive, red) poor quality of life. **A** The training set; **B** The validation set. Probability density curves were plotted against predicted probability. Among the patients with poor quality of life (green), the peak of its curve was located at a high level of predicted probability, while the peak of its curve was located at a low level of predicted probability among the patients without poor quality of life (red), suggesting that the nomogram had favorable discriminative ability

**Fig. 4:**
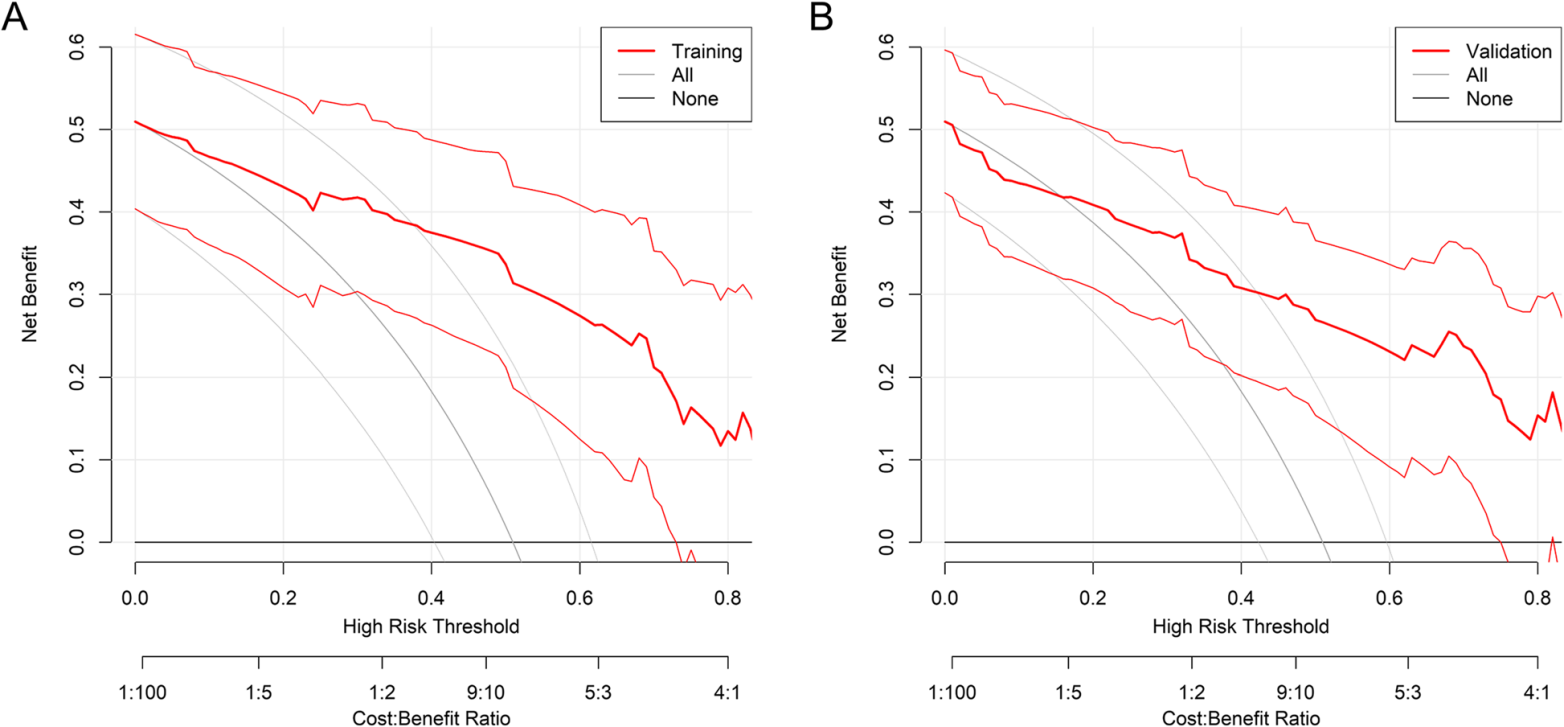
Decision curve analysis of the nomogram. **A** The benefit in the training set; **B** The benefit in the validation set. The thick solid gray line indicates treated-for-all scheme and the thin solid gray lines indicate 95% confident interval of treated-for-all scheme. The horizontal black line indicates treated-for-none scheme. The thick solid red line indicates the decision curve with 95% confident interval of it (the thin solid red lines). Cost:benefit ratio was presented in the below of each decision curve. The decision curves are a significant distance from the two reference lines (the treat-for-all scheme and the treat-for-none scheme), suggesting that the nomogram performed well in clinical setting in both the training and validation sets

Area under the receiver operating characteristic curve (AUROC) was 0.90 (95% CI: 0.84–0.96) in the training set and 0.85 (95% CI: 0.78–0.93) in the validation set, and the corresponding discrimination slopes were 0.50 (95% CI: 0.41–0.58) and 0.44 (95% CI: 0.33–0.56), respectively. The probability density curves were displayed for the training (Fig. 3A) and validation (Fig. 3B) sets. Among the patients with poor quality of life (positive events), the peak of its curve was seen at a high level of predicted probability. However, among the patients without poor quality of life (negative events), the peak of its curve was situated at a low level of predicted probability. The above findings all suggested that the nomogram had a good capacity for discrimination. The nomogram’s calibration curves presented high agreement between the predicted and observed probability of poor quality of life in the training and validation sets. The treat-for-all and the treat-for-none lines were distant from the decision curves, indicating that the nomogram also showed favorable clinical usefulness in both the training (Fig. 4A) and validation (Fig. 4B) sets. As a result, the nomogram had considerable discriminative and calibrating performance.

Additional performance metrics for the two groups are shown in Table 2. In detail, the Brier score was 0.13 in the training set and 0.16 in the validation set, both of which were lower than 0.25, suggesting favorable overall prediction performance of the nomogram. In addition, the corresponding Brier_scaled_ scores were 48.96 and 34.77%, respectively. The accuracy was 83.65% in the training set and 79.81% in the validation set, and the corresponding specificities were 82.35 and 74.51%, respectively, and the sensitivities were both 84.91%.

**Table 2:** Predictive performance of the nomogram to predict relatively poor quality of life among patients with spine metastases in the training and validation sets

Predictive metrics	Training set	Validation set
Brier	0.13	0.16
Brier_scaled_	48.96%	34.77%
AUROC (95% CI)	0.90 (0.84–0.96)	0.85 (0.78–0.93)
Discrimination slope (95% CI)	0.50 (0.41–0.58)	0.44 (0.33–0.56)
Accuracy	83.65%	79.81%
Sensitivity	84.91%	84.91%
Specificity	82.35%	74.51%
Recall	84.91%	84.91%
Youden index	1.67	1.59
Threshold	44.24%	32.07%

*AUROC* area under the receiver operating characteristic curve, *CI* confident interval

### Risk stratification based on the nomogram

Patients were categorized into a low-risk group and a high-risk group in terms of the threshold. In the study, the best cut-off point that used for risk stratification among the entire patients was the average of the thresholds in the training (44.24%) and validation (32.07%) groups. As a result, 40.00% was chosen as the cut-off point to facilitate clinical practice. Low-risk patients were those with a predicted probability of less than 40.0%, while high-risk patients were those with a predicted probability of 40.0% or more. When compared to patients in the low-risk group, patients in the high-risk group were more than four times as likely to have a very poor quality of life (82.18% vs. 21.50%, *P* < 0.001, Table 3).

**Table 3:** Risk group classification of spine metastasis patients based on the nomogram

Risk groups	Predicted probability	Observed probability (***n*** = 208)	P ^**a**^
Low-risk group (< 40.00%)	16.08%	21.50% (23/107)	< 0.001
High-risk group (≥40.00%)	82.53%	82.18% (83/101)	

^a^ indicates P was obtained from Chi-square test for a comparison of observed probability between the low and high-risk groups

**Fig. 5:**
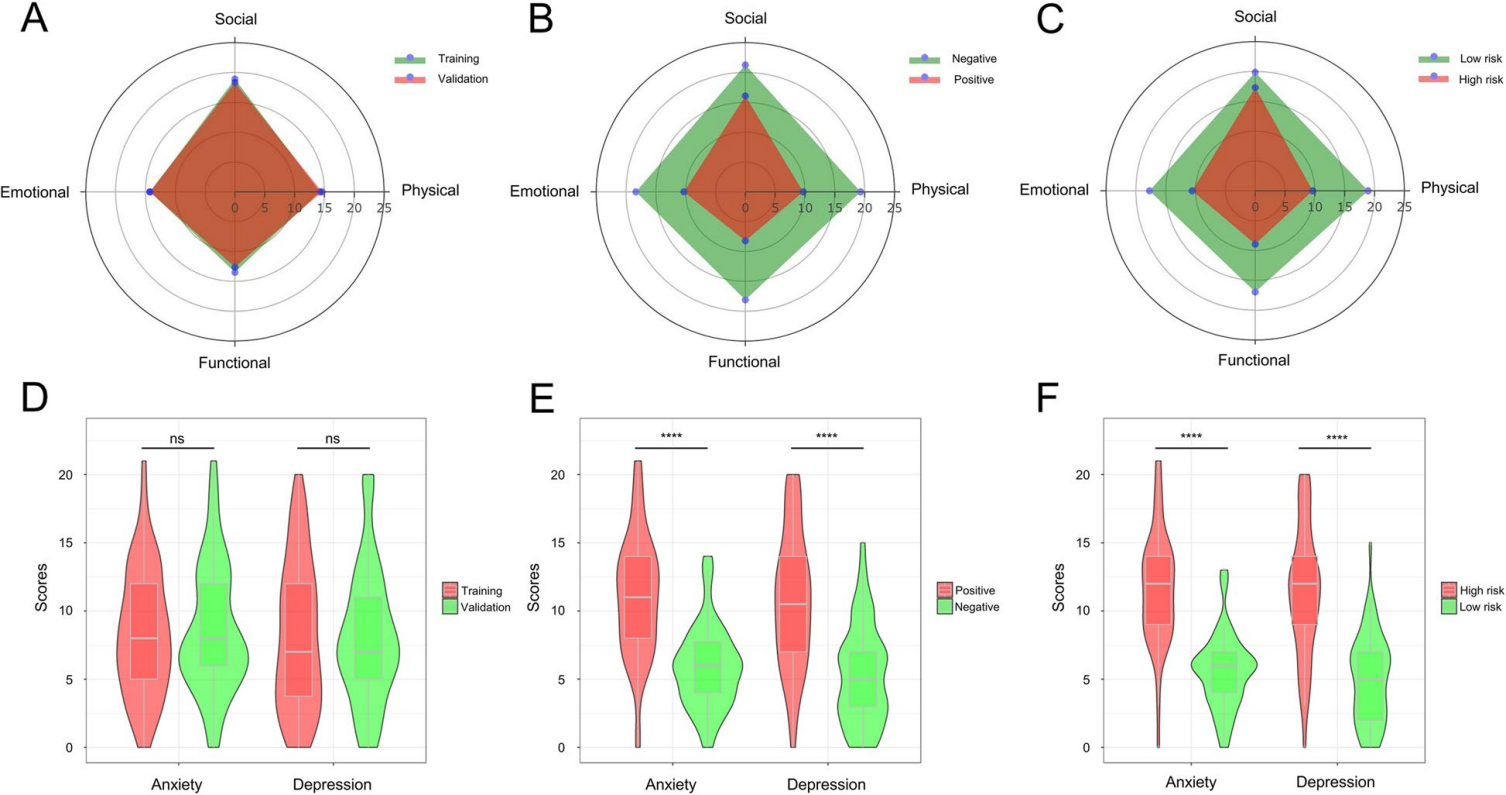
Subgroup analysis based on the radar charts and violin plots. **A** The radar chart to describe the distribution of the four subscale scores among the training (green) vs. validation (red) groups; **B** The radar chart to describe the distribution of the four subscale scores among the negative (green) vs. positive (red) events; **C** The radar chart to describe the distribution of the four subscale scores among the low-risk (green) vs. high-risk (red) groups. The four subscales include the physical well-being score, social well-being score, emotional well-being score, and functional well-being score. **D** Violin plot for anxiety and depression scores between the training (red) and validation (green) groups. **E** Violin plot for anxiety and depression scores between the negative (green) vs. positive (red) events. **F** Violin plot for anxiety and depression scores between the low-risk (green) vs. high-risk (red) groups. Positive event indicates patients with poor quality of life; Negative event indicates patients without poor quality of life. “ns” indicates no significance; **** indicates *P* < 0.0001 (the Wilcoxon two-sample test)

Radar charts were used to visualize the four subscales of the FACT-G and determined the distribution between the training and validation sets (Fig. 5A), the negative and positive groups (Fig. 5B), and the low-risk and high-risk groups (Fig. 5C). Figure 5A indicates that the four subscales of FACT-G—the physical well-being, social well-being, emotional well-being, and functional well-being—were similarly distributed between the training and validation sets, which could be severed as a negative control for the nomogram stratification. Figure 5B indicates patients with relatively poor quality of life (positive events) had obviously lower scores as compared to patients without relatively poor quality of life (negative events), and this finding could be severed as a positive control for the nomogram stratification. Figure 5C indicates a very similar distribution to Fig. 5B, denoting favorable risk stratification based on the nomogram because patients in the high-risk group had distinct lower scores as compared to patients in the low-risk group.

Additionally, violine plots were utilized to illustrate the levels of anxiety and depression scores in above subgroups. The anxiety and depression scores were both comparable between the training and validation sets (Fig. 5D). Patients with positive events (poor quality of life) had significantly higher anxiety and depression scores as compared to patients with negative events (Fig. 5E, *P* < 0.0001), indicating patients suffering from poor quality of life also experienced more severe anxious and depressive symptoms. Figure 5F shows that patients in the high-risk group had significant higher anxiety and depression scores than patients in the low-risk group (*P* < 0.0001), indicating the nomogram not only achieved excellent stratification of risk probability of poor quality of life, but good stratification of mental health status.

## Discussion

This study offered a nomogram to predict and stratify quality of life especially among advanced cancer patients with spine metastases, and this nomogram was also internally validated using the bootstrap method and evaluated using discrimination and calibration. The assessment of predictive performance, which took into account the Brier score, AUROC, discrimination slope, accuracy, Recall, and Youden index, demonstrated that the nomogram had favorable discriminative and calibrating ability. A web-calculator was created to facilitate clinical utility. Everyone had access to this web calculator once their electronic devices connected to the Internet. Additionally, the web calculator was simple to use because it only required selecting and filling the appropriate items and clicking “Predict” option to obtain the predicted risk probability. Patients were classified into a low-risk group and a high-risk group based on the nomogram. Patients in the high-risk group suffered from poorer quality of life and more serious anxiety and depression than patients in the low-risk group. Thus, the nomogram achieved both favorable stratification of quality of life and mental health status among advanced cancer patients with spine metastases.

The average FACT-G score was only 60.32 ± 20.41, showing that individuals with spine metastases lived with very poor quality of life as compared to general populations. This number was even lower than that in general cancer patients since their FACT-G score was approximate 70.00 [22]. Besides, this study found that about 50.00% of patients experienced skeptical or confirmed anxiety or depression, which suggested that the mental health status of those patients was far from satisfaction. As a result, a prediction model to assess the quality of life using mental condition would be great helpful for clinical therapeutic strategies. Patient’s mental health status and quality of life could be both evaluated and accessible to doctors. Notably, as far as author’s awareness, this nomogram was the first prediction model to predict and categorize quality of life, particularly in advanced cancer patients with spine metastases. Actually, due to population heterogeneity, the definition of poor quality of life did not have widely accepted criteria. In a previous study, Sehlen et al. [23] used the FACT-G sum score and set the cutoff score at 70.0 among patients with head-and-neck cancer. The general condition of those patients was good since the vast majority of patients (97.0%) did not have metastasis, 78.3% patients were treated with surgery, and 45.6% patients were in N0 stage. Nonetheless, in our analysis, all patients had spinal metastatic disease and 56.73% of patients had visceral metastasis. Therefore, the cutoff value used in the study conducted by Sehlen et al. [23] might not be applicable in our study, because the patients in our study had more serious health problems as compared to patients in the study of Sehlen et al. [23]. Of note, when no cut-off value was available for this specific group, the best way to determine a threshold is to use the median of the continuous variable. Therefore, the present study defined that a FACT-G score of less than 60, the median of FACT-G scores among the entire cohort of patients, represented relatively poor quality of life.

The nomogram included six variables, including ECOG score, having an uncompleted life goal, preference for eating vegetables, chemotherapy, anxiety status, and depression status, and those variables were widely available during hospitalization. The majority of the aforementioned variables had been verified by previous studies. For instance, Daly et al. [7] had already pointed out that in general incurable cancer patients, a higher ECOG score was independently associated with poorer quality of life scores. Zhang et al. [24] demonstrated that preference for eating vegetables could be a protective factor for anxiety and depression in general university students. Regarding chemotherapy, it negatively affected spine metastases patient’s quality of life possibly as a result of chemotherapy-induced side effects [25]. Taira et al. [26] concluded that adjuvant chemotherapy had a negative impact on quality of life among patients with breast cancer, and the effects could last for at least 12 months but were not noticeable at 36 months. Thus, it was important to identify and address adverse drug reaction by performing proper approaches to promote judicious use of chemical drugs [27]. Decreased anxiety and depression were closely associated with increased quality of life among cancer patients [28], and this founding supported our present study. In general, it should be noted that establishing models based on the well-known findings may help prediction models become more accurate and acceptable among researchers.

A prediction model to assess the quality of life would be great helpful for doctors to make appropriate patient’s selection and timely conduct effective interventions. Therefore, several prediction models were developed to predict the quality of life among patients treated with lumbar spine surgery [29], young adult patients with stroke [30], ICU survivors [31], patients undergoing haemodialysis [32], pulmonary tuberculosis patients [33], older men living alone [34], and multiple sclerosis patients [35]. However, prediction models for cancer patients to assess quality of life were not commonly reported. After carefully reviewing literature, several articles were identified as focusing on development of model to predict quality of life among cancer patients. Revesz et al. [36] designed and internally validated prediction models to assess health-related quality of life among colorectal cancer patients. The prediction models included a multitude of variables, including non-modifiable predictors, such as age, sex, socio-economic status, time since diagnosis, tumor stage, chemotherapy, radiotherapy, stoma, micturition, chemotherapy-related, stoma-related and gastrointestinal complaints, comorbidities, social inhibition/negative affectivity, and working status, and modifiable predictors, such as body mass index, physical activity, smoking, meat consumption, anxiety/depression, and pain. It might be challenging to employ the prediction models in clinical practice since it was not easy for doctors to collect so many variables. Formica et al. [37] proposed a nomogram to predict health-related quality of life among colorectal cancer patients, and the nomogram included only age, sex, and body mass index, but the C-statistics of the nomogram was only 0.67, indicating that its predictive power was not very strong. The C-statistics for the prediction nomogram put forth in this study could reach 0.90 in the training set and 0.85 in the validation set. Our nomogram was especially designed for spine metastases patients and only included six common variables. In addition, a web-calculator was created to promote clinical practice, and it would be very convenient for doctors and patients to use.

## Limitations

However, we acknowledged the limitations of the study. First of all, the heterogeneity of the sample might lead to bias since this is a single center study, despite the fact that the data were prospectively collected. Secondly, the study did not analyze effective supportive strategies, such as nutrition and pain alleviation, and after introducing those variables the prediction performance of the nomogram might be further improved. However, this study analyzed as many as twenty-five potential risk factors and the predictive effectiveness of the nomogram was favorable based on the evaluation metrics. Thirdly, the long-term condition of quality of life was not evaluated in the study. Lastly, external validation of the present nomogram was not conducted in the study, and thus the generalization of the nomogram was still unknown. Therefore, although the nomogram had favorable prediction performance, long-term follow up and external validation were still warranted in a large independent cohort.

## Conclusions

This study suggests a nomogram that can be applied as a practical clinical tool to forecast and categorize the quality of life among patients with spine metastases. Additionally, patients with poor quality of life experience more severe anxiety and depression. Effective interventions should be carried out as soon as possible, especially for patients in the high-risk group, to improve their quality of life and mental health condition.

## Supplementary Information

**Additional file 1.**

**Additional file 2.**

**Additional file 3.**

**Additional file 4.**

**Additional file 5.**

**Additional file 6.**

**Additional file 7.**

**Additional file 8.**

**Additional file 9.**

**Additional file 10.**

## Data Availability

The data are available under reasonable request to the corresponding author.

## Abbreviations

AUROC: Area under the receiver operating characteristic curve
CI: Confident interval
ECOG: Eastern cooperative oncology group
FACT-G: Functional assessment of cancer therapy-general
SD: Standard deviation

## References

[1] Sung H, Ferlay J, Siegel RL, Laversanne M, Soerjomataram I, Jemal A (2021). Global Cancer statistics 2020: GLOBOCAN estimates of incidence and mortality worldwide for 36 cancers in 185 countries. CA Cancer J Clin.

[2] Jaipanya P, Chanplakorn P (2022). Spinal metastasis: narrative reviews of the current evidence and treatment modalities. J Int Med Res.

[3] Giammalva GR, Ferini G, Torregrossa F, Brunasso L, Musso S, Benigno UE, et al. The palliative Care in the Metastatic Spinal Tumors. A Systematic Review on the Radiotherapy and Surgical Perspective Life (Basel). 2022;12;12(4). 10.3390/life12040571 PMC9032747 35455062

[4] Parker PA, Baile WF, de Moor C, Cohen L (2003). Psychosocial and demographic predictors of quality of life in a large sample of cancer patients. Psychooncology..

[5] Nipp RD, Fuchs G, El-Jawahri A, Mario J, Troschel FM, Greer JA (2018). Sarcopenia is associated with quality of life and depression in patients with advanced Cancer. Oncologist..

[6] Jacob J, Palat G, Verghese N, Chandran P, Rapelli V, Kumari S (2019). Health-related quality of life and its socio-economic and cultural predictors among advanced cancer patients: evidence from the APPROACH cross-sectional survey in Hyderabad-India. BMC Palliative Care.

[7] Daly LE, Dolan RD, Power DG, Ni Bhuachalla E, Sim W, Cushen SJ (2020). Determinants of quality of life in patients with incurable cancer. Cancer..

[8] Barzilai O, Versteeg AL, Goodwin CR, Sahgal A, Rhines LD, Sciubba DM (2019). Association of neurologic deficits with surgical outcomes and health-related quality of life after treatment for metastatic epidural spinal cord compression. Cancer..

[9] Bernard F, Lemee JM, Lucas O, Menei P (2017). Postoperative quality-of-life assessment in patients with spine metastases treated with long-segment pedicle-screw fixation. J Neurosurg Spine.

[10] Tomita K, Kawahara N, Kobayashi T, Yoshida A, Murakami H, Akamaru T (2001). Surgical strategy for spinal metastases. Spine..

[11] Tokuhashi Y, Matsuzaki H, Oda H, Oshima M, Ryu J (2005). A revised scoring system for preoperative evaluation of metastatic spine tumor prognosis. Spine (Phila Pa 1976).

[12] Lei M, Li J, Liu Y, Jiang W, Liu S, Zhou S. Who are the best candidates for Decompressive surgery and spine stabilization in patients with metastatic spinal cord compression?: a new scoring system. Spine (Phila Pa 1976). 2016;41(18):1469–1476. 10.1097/BRS.0000000000001538 PMC5001136 26937605

[13] Cui Y, Lei M, Pan Y, Lin Y, Shi X (2020). Scoring algorithms for predicting survival prognosis in patients with metastatic spinal disease: the current status and future directions. Clin Spine Surg.

[14] Yu CL, Fielding R, Chan CL, Tse VK, Choi PH, Lau WH (2000). Measuring quality of life of Chinese cancer patients: a validation of the Chinese version of the functional assessment of Cancer therapy-general (FACT-G) scale. Cancer..

[15] Tang Y, Qu J, Wu J, Liu H, Chu T, Xiao J (2016). Effect of surgery on quality of life of patients with spinal metastasis from non-small-cell lung Cancer. J Bone Joint Surg Am.

[16] Zigmond AS, Snaith RP (1983). The hospital anxiety and depression scale. Acta Psychiatr Scand.

[17] Poyraz BC, Poyraz CA, Olgun Y, Gurel O, Alkan S, Ozdemir YE (2021). Psychiatric morbidity and protracted symptoms after COVID-19. Psychiatry Res.

[18] Zhang J, Xiao LH, Pu SY, Liu Y, He JJ, Wang K (2021). Can we reliably identify the pathological outcomes of Neoadjuvant chemotherapy in patients with breast Cancer? Development and validation of a logistic regression nomogram based on preoperative factors. Ann Surg Oncol.

[19] Wu J, Zhang H, Li L, Hu M, Chen L, Xu B (2020). A nomogram for predicting overall survival in patients with low-grade endometrial stromal sarcoma: a population-based analysis. Cancer Communications.

[20] Steyerberg EW, Vickers AJ, Cook NR, Gerds T, Gonen M, Obuchowski N (2010). Assessing the performance of prediction models: a framework for traditional and novel measures. Epidemiology..

[21] Fitzgerald M, Saville BR, Lewis RJ (2015). Decision curve analysis. JAMA..

[22] Franciosi V, Maglietta G, Degli Esposti C, Caruso G, Cavanna L, Berte R (2019). Early palliative care and quality of life of advanced cancer patients-a multicenter randomized clinical trial. Ann Palliat Med.

[23] Sehlen S, Hollenhorst H, Lenk M, Schymura B, Herschbach P, Aydemir U (2002). Only sociodemographic variables predict quality of life after radiography in patients with head-and-neck cancer. Int J Radiat Oncol Biol Phys.

[24] Zhang L, Zhao S, Lin Q, Song M, Wu S, Zheng H (2021). Algorithms to predict anxiety and depression Among University students in China after analyzing lifestyles and sport habits. Neuropsychiatr Dis Treat.

[25] Trendowski MR, Lusk CM, Ruterbusch JJ, Seaton R, Simon MS, Greenwald MK (2021). Chemotherapy-induced peripheral neuropathy in African American cancer survivors: risk factors and quality of life outcomes. Cancer Med.

[26] Taira N, Sawaki M, Uemura Y, Saito T, Baba S, Kobayashi K (2021). Health-related quality of life with Trastuzumab monotherapy versus Trastuzumab plus standard chemotherapy as adjuvant therapy in older patients with HER2-positive breast Cancer. J Clin Oncol.

[27] Krishnarajan D, Sivasakthi K, Ariyamol R, Kumar DN, Varghese S (2021). A prospective observational study of chemotherapy-induced adverse drug reaction and the quality of life in cancer patients in a tertiary care hospital. J Cancer Res Ther.

[28] Castro-Figueroa EM, Torres-Blasco N, Rosal MC, Jimenez JC, Castro-Rodriguez WP, Gonzalez-Lorenzo M (2021). Brief report: Hispanic Patients' trajectory of Cancer symptom burden, depression, anxiety, and quality of life. Nurs Rep.

[29] Lubelski D, Feghali J, Nowacki AS, Alentado VJ, Planchard R, Abdullah KG (2021). Patient-specific prediction model for clinical and quality-of-life outcomes after lumbar spine surgery. J Neurosurg Spine..

[30] Yoon S, Kim HY, Kim SR (2021). A prediction model of health-related quality of life in young adult patients with stroke. J Clin Nurs.

[31] Wubben N, van den Boogaard M, Ramjith J, Bisschops LLA, Frenzel T, van der Hoeven JG (2021). Development of a practically usable prediction model for quality of life of ICU survivors: a sub-analysis of the MONITOR-IC prospective cohort study. J Crit Care.

[32] Jung HM, Kim HY (2020). A health-related quality of life model for patients undergoing haemodialysis. J Clin Nurs.

[33] Jang KS, Jeon GS (2017). Prediction model for health-related quality of life in hospitalized patients with pulmonary tuberculosis. J Korean Acad Nurs.

[34] Kim SJ, Jeon GS (2020). Predictive model for quality of life of the older men living alone. J Korean Acad Nurs.

[35] Gil-Gonzalez I, Perez-San-Gregorio MA, Conrad R, Martin-Rodriguez A (2021). Predicting improvement of quality of life and mental health over 18-months in multiple sclerosis patients. Mult Scler Relat Disord.

[36] Revesz D, van Kuijk SMJ, Mols F, van Duijnhoven FJB, Winkels RM, Hoofs H (2020). Development and internal validation of prediction models for colorectal cancer survivors to estimate the 1-year risk of low health-related quality of life in multiple domains. BMC Med Inform Decis Mak.

[37] Formica V, Nardecchia A, Morelli C, Lucchetti J, Giuliano G, Renzi N (2021). Health-related quality of life in patients with advanced colorectal cancer: a predictive nomogram including BMI, sex and age. Ann Palliat Med..

