# Supplementary material for: A novel nomogram to stratify quality of life among advanced cancer patients with spinal metastatic disease after examining demographics, dietary habits, therapeutic interventions, and mental health status
Source: BMC Cancer. 2022 Nov 23;22:1205. doi: 10.1186/s12885-022-10294-z (PMC9694561; doi:10.1186/s12885-022-10294-z)
Supplement: Supplementary file 1 — Additional file 1. [file 12885_2022_10294_MOESM1_ESM.docx]

**Additional file 1: Supplementary Fig. 1.** Area under the receiver operating characteristic curve (AUROC) of using the nomogram to predict poor quality of life. (A) The training set; (B) The validation set. The AUROC was 0.90 (95% CI: 0.84-0.96) in the training set and 0.85 (95% CI: 0.78-0.93) in the validation set, both of which were considered ideal.
