# Supplementary material for: A novel nomogram to stratify quality of life among advanced cancer patients with spinal metastatic disease after examining demographics, dietary habits, therapeutic interventions, and mental health status
Source: BMC Cancer. 2022 Nov 23;22:1205. doi: 10.1186/s12885-022-10294-z (PMC9694561; doi:10.1186/s12885-022-10294-z)
Supplement: Supplementary file 2 — Additional file 2. [file 12885_2022_10294_MOESM2_ESM.docx]

**Additional file 2: Supplementary Fig. 2.** Box plots of predicted probabilities among patients without (negative) and with (positive) poor quality of life. (A) The training set (discrimination slope = 0.50, 95% CI: 0.41-0.58); (B) The validation set (discrimination slope = 0.44, 95% CI: 0.33-0.56). The discrimination slope was calculated as the difference between the mean predicted probability with and without poor quality of life (solid dots indicate means).
