# Supplementary material for: A novel nomogram to stratify quality of life among advanced cancer patients with spinal metastatic disease after examining demographics, dietary habits, therapeutic interventions, and mental health status
Source: BMC Cancer. 2022 Nov 23;22:1205. doi: 10.1186/s12885-022-10294-z (PMC9694561; doi:10.1186/s12885-022-10294-z)
Supplement: Supplementary file 3 — Additional file 3. [file 12885_2022_10294_MOESM3_ESM.docx]

**Additional file 3: Supplementary Fig. 3.** Calibration curves of using the nomogram to predict poor quality of life. (A) The training set; (B) The validation set. Calibration curves were plotted by predicted probability against observed probability. The gray dotted line indicates the ideal reference line where predicted probability could perfectly match the observed probability. The black dotted line indicates the ideal calibration of the nomogram, the red solid line indicates the original calibration of the nomogram, and the blue solid line indicates the bias-corrected calibration of the nomogram. The closer the solid line is to the gray dotted line, the better calibrating ability the nomogram has.
