# Supplementary material for: A novel nomogram to stratify quality of life among advanced cancer patients with spinal metastatic disease after examining demographics, dietary habits, therapeutic interventions, and mental health status
Source: BMC Cancer. 2022 Nov 23;22:1205. doi: 10.1186/s12885-022-10294-z (PMC9694561; doi:10.1186/s12885-022-10294-z)
Supplement: Supplementary file 4 — Additional file 4. [file 12885_2022_10294_MOESM4_ESM.docx]

| **Additional file 4.**  **Supplementary table 1.** Subgroup analysis of patients stratified by chemotherapy. | | | | |
| --- | --- | --- | --- | --- |
| Clinical characteristics | Overall | Chemotherapy | | P |
|  |  | No | Yes |  |
| n | 208 | 82 | 126 |  |
| Age (mean (SD), years) | 58.74 (12.00) | 57.49 (14.85) | 59.56 (9.71) | 0.226 |
| Sex (male/female, %) | 107/101 (51.4/48.6) | 42/40 (51.2/48.8) | 65/61 (51.6/48.4) | 1.000 |
| Nationality (han/minorities, %) | 201/7 (96.6/3.4) | 81/1 (98.8/1.2) | 120/6 (95.2/4.8) | 0.322 |
| Marital status (married/single, %) | 194/14 (93.3/6.7) | 76/6 (92.7/7.3) | 118/8 (93.7/6.3) | 1.000 |
| Education (%) |  |  |  | 0.042 |
| Primary education | 74 (35.6) | 34 (41.5) | 40 (31.7) |  |
| Senior high school | 73 (35.1) | 32 (39.0) | 41 (32.5) |  |
| University or above | 61 (29.3) | 16 (19.5) | 45 (35.7) |  |
| Caregivers (%) |  |  |  | 0.510 |
| Spouse | 135 (64.9) | 52 (63.4) | 83 (65.9) |  |
| Other family members | 39 (18.8) | 18 (22.0) | 21 (16.7) |  |
| Support workers | 10 (4.8) | 5 (6.1) | 5 (4.0) |  |
| No caregivers | 24 (11.5) | 7 (8.5) | 17 (13.5) |  |
| Preference to eat vegetables (no/yes, %) | 28/180 (13.5/86.5) | 6/76 (7.3/92.7) | 22/104 (17.5/82.5) | 0.059 |
| Preference to eat roasted food (no/yes, %) | 188/20 (90.4/9.6) | 74/8 (90.2/9.8) | 114/12 (90.5/9.5) | 1.000 |
| Smoking status (%) |  |  |  | 0.290 |
| No | 119 (57.2) | 43 (52.4) | 76 (60.3) |  |
| Quitting smoking | 49 (23.6) | 24 (29.3) | 25 (19.8) |  |
| Current smoking | 40 (19.2) | 15 (18.3) | 25 (19.8) |  |
| Drinking status (%) |  |  |  | 0.757 |
| No | 153 (73.6) | 58 (70.7) | 95 (75.4) |  |
| Quitting drinking | 39 (18.8) | 17 (20.7) | 22 (17.5) |  |
| Current drinking | 16 (7.7) | 7 (8.5) | 9 (7.1) |  |
| Hypertension (no/yes, %) | 157/51 (75.5/24.5) | 61/21 (74.4/25.6) | 96/30 (76.2/23.8) | 0.897 |
| Diabetes (no/yes, %) | 188/20 (90.4/9.6) | 74/8 (90.2/9.8) | 114/12 (90.5/9.5) | 1.000 |
| Coronary heart disease (no/yes, %) | 192/16 (92.3/7.7) | 76/6 (92.7/7.3) | 116/10 (92.1/7.9) | 1.000 |
| Time since knowing cancer diagnosis (%) |  |  |  | 0.015 |
| < 3 months | 37 (17.8) | 21 (25.6) | 16 (12.7) |  |
| ≧3 months and < 6 months | 21 (10.1) | 12 (14.6) | 9 (7.1) |  |
| ≧6 months and < 12 months | 21 (10.1) | 6 (7.3) | 15 (11.9) |  |
| ≧12 months | 129 (62.0) | 43 (52.4) | 86 (68.3) |  |
| Primary cancer type (%) |  |  |  | 0.043 |
| Lung cancer | 119 (57.2) | 38 (46.3) | 81 (64.3) |  |
| Liver cancer | 10 (4.8) | 5 (6.1) | 5 (4.0) |  |
| Gastrointestinal cancer | 16 (7.7) | 7 (8.5) | 9 (7.1) |  |
| Breast cancer | 20 (9.6) | 7 (8.5) | 13 (10.3) |  |
| Others | 43 (20.7) | 25 (30.5) | 18 (14.3) |  |
| Visceral metastasis (no/yes, %) | 118/90 (56.7/43.3) | 60/22 (73.2/26.8) | 58/68 (46.0/54.0) | <0.001 |
| Surgery for primary cancer site (%) |  |  |  | 0.334 |
| Open surgery | 41 (19.7) | 14 (17.1) | 27 (21.4) |  |
| Minimally invasive surgery | 43 (20.7) | 21 (25.6) | 22 (17.5) |  |
| None | 124 (59.6) | 47 (57.3) | 77 (61.1) |  |
| Surgery for spine metastasis (%) |  |  |  | 0.294 |
| Open surgery | 33 (15.9) | 17 (20.7) | 16 (12.7) |  |
| Minimally invasive surgery | 114 (54.8) | 43 (52.4) | 71 (56.3) |  |
| None | 61 (29.3) | 22 (26.8) | 39 (31.0) |  |
| Radiotherapy (no/yes, %) | 82/126 (39.4/60.6) | 43/39 (52.4/47.6) | 39/87 (31.0/69.0) | 0.003 |
| Chemotherapy (no/yes, %) | 82/126 (39.4/60.6) | 82/0 (100.0/0.0) | 0/126 (0.0/100.0) | <0.001 |
| Economic burden due to cancer treatments (%) | |  |  | 0.002 |
| None | 6 (2.9) | 3 (3.7) | 3 (2.4) |  |
| Mild | 22 (10.6) | 15 (18.3) | 7 (5.6) |  |
| Moderate | 67 (32.2) | 16 (19.5) | 51 (40.5) |  |
| Severe | 113 (54.3) | 48 (58.5) | 65 (51.6) |  |
| Having an uncompleted life goal (no/yes, %) | 50/158 (24.0/76.0) | 28/54 (34.1/65.9) | 22/104 (17.5/82.5) | 0.010 |
| ECOG scores (%) |  |  |  | 0.004 |
| 0 | 14 (6.7) | 10 (12.2) | 4 (3.2) |  |
| 1 | 71 (34.1) | 34 (41.5) | 37 (29.4) |  |
| 2 | 62 (29.8) | 24 (29.3) | 38 (30.2) |  |
| 3 | 24 (11.5) | 5 (6.1) | 19 (15.1) |  |
| 4 | 37 (17.8) | 9 (11.0) | 28 (22.2) |  |
| Anxiety (%) |  |  |  | 0.149 |
| No | 99 (47.6) | 45 (54.9) | 54 (42.9) |  |
| Skeptical | 43 (20.7) | 17 (20.7) | 26 (20.6) |  |
| Yes | 66 (31.7) | 20 (24.4) | 46 (36.5) |  |
| Depression (%) |  |  |  | 0.209 |
| No | 107 (51.4) | 48 (58.5) | 59 (46.8) |  |
| Skeptical | 40 (19.2) | 15 (18.3) | 25 (19.8) |  |
| Yes | 61 (29.3) | 19 (23.2) | 42 (33.3) |  |
| Relatively poor quality of life (no/yes, %) | 102/106 (49.0/51.0) | 54/28 (65.9/34.1) | 48/78 (38.1/61.9) | <0.001 |
| FACT-G score (mean (SD)) | 60.32 (20.41) | 66.43 (20.69) | 56.35 (19.30) | <0.001 |
| Physical well-being (mean (SD)) | 14.41 (7.22) | 16.76 (7.12) | 12.89 (6.89) | <0.001 |
| Social well-being (mean (SD)) | 18.62 (5.82) | 19.72 (5.28) | 17.90 (6.06) | 0.027 |
| Emotional well-being (mean (SD)) | 14.24 (5.70) | 15.43 (5.88) | 13.47 (5.46) | 0.015 |
| Functional well-being (mean (SD)) | 13.05 (7.14) | 14.52 (6.86) | 12.10 (7.18) | 0.016 |
| *Abbreviations: ECOG eastern cooperative oncology group; FACT-G functional assessment of cancer therapy-general; SD standard deviation.* | | | | |
