# Supplementary material for: A novel nomogram to stratify quality of life among advanced cancer patients with spinal metastatic disease after examining demographics, dietary habits, therapeutic interventions, and mental health status
Source: BMC Cancer. 2022 Nov 23;22:1205. doi: 10.1186/s12885-022-10294-z (PMC9694561; doi:10.1186/s12885-022-10294-z)
Supplement: Supplementary file 5 — Additional file 5. [file 12885_2022_10294_MOESM5_ESM.docx]

| **Additional file 5.**  **Supplementary table 2.** Subgroup analysis of patients stratified by depression. | | | | | |
| --- | --- | --- | --- | --- | --- |
| Clinical characteristics | Overall | Depression | | | P |
|  |  | No | Skeptical | Yes |  |
| n | 208 | 107 | 40 | 61 |  |
| Age (mean (SD), years) | 58.74 (12.01) | 55.49 (13.54) | 60.00 (9.63) | 63.62 (8.29) | <0.001 |
| Sex (male/female, %) | 107/101 (51.4/48.6) | 48/59 (44.9/55.1) | 22/18 (55.0/45.0) | 37/24 (60.7/39.3) | 0.127 |
| Nationality (han/minorities, %) | 201/7 (96.6/3.4) | 100/7 (93.5/6.5) | 40/0 (100.0/0.0) | 61/0 (100.0/0.0) | 0.033 |
| Marital status (married/single, %) | 194/14 (93.3/6.7) | 99/8 (92.5/7.5) | 36/4 (90.0/10.0) | 59/2 (96.7/3.3) | 0.380 |
| Education (%) |  |  |  |  | 0.002 |
| Primary education | 74 (35.6) | 26 (24.3) | 14 (35.0) | 34 (55.7) |  |
| Senior high school | 73 (35.1) | 43 (40.2) | 14 (35.0) | 16 (26.2) |  |
| University or above | 61 (29.3) | 38 (35.5) | 12 (30.0) | 11 (18.0) |  |
| Caregivers (%) |  |  |  |  | 0.029 |
| Spouse | 135 (64.9) | 74 (69.2) | 25 (62.5) | 36 (59.0) |  |
| Other family members | 39 (18.8) | 18 (16.8) | 5 (12.5) | 16 (26.2) |  |
| Support workers | 10 (4.8) | 7 (6.5) | 0 (0.0) | 3 (4.9) |  |
| No caregivers | 24 (11.5) | 8 (7.5) | 10 (25.0) | 6 (9.8) |  |
| Preference to eat vegetables (no/yes, %) | 28/180 (13.5/86.5) | 7/100 (6.5/93.5) | 13/27 (32.5/67.5) | 8/53 (13.1/86.9) | <0.001 |
| Preference to eat roasted food (no/yes, %) | 188/20 (90.4/9.6) | 94/13 (87.9/12.1) | 40/0 (100.0/0.0) | 54/7 (88.5/11.5) | 0.071 |
| Smoking status (%) |  |  |  |  | 0.458 |
| No | 119 (57.2) | 67 (62.6) | 20 (50.0) | 32 (52.5) |  |
| Quitting smoking | 49 (23.6) | 24 (22.4) | 11 (27.5) | 14 (23.0) |  |
| Current smoking | 40 (19.2) | 16 (15.0) | 9 (22.5) | 15 (24.6) |  |
| Drinking status (%) |  |  |  |  | 0.475 |
| No | 153 (73.6) | 76 (71.0) | 29 (72.5) | 48 (78.7) |  |
| Quitting drinking | 39 (18.8) | 23 (21.5) | 9 (22.5) | 7 (11.5) |  |
| Current drinking | 16 (7.7) | 8 (7.5) | 2 (5.0) | 6 (9.8) |  |
| Hypertension (no/yes, %) | 157/51 (75.5/24.5) | 81/26 (75.7/24.3) | 29/11 (72.5/27.5) | 47/14 (77.0/23.0) | 0.871 |
| Diabetes (no/yes, %) | 188/20 (90.4/9.6) | 96/11 (89.7/10.3) | 36/4 (90.0/10.0) | 56/5 (91.8/8.2) | 0.904 |
| Coronary heart disease (no/yes, %) | 192/16 (92.3/7.7) | 101/6 (94.4/5.6) | 38/2 (95.0/5.0) | 53/8 (86.9/13.1) | 0.166 |
| Time since knowing cancer diagnosis (%) | |  |  |  | 0.568 |
| < 3 months | 37 (17.8) | 21 (19.6) | 4 (10.0) | 12 (19.7) |  |
| ≧3 months and < 6 months | 21 (10.1) | 10 (9.3) | 6 (15.0) | 5 (8.2) |  |
| ≧6 months and < 12 months | 21 (10.1) | 11 (10.3) | 6 (15.0) | 4 (6.6) |  |
| ≧12 months | 129 (62.0) | 65 (60.7) | 24 (60.0) | 40 (65.6) |  |
| Primary cancer type (%) |  |  |  |  | <0.001 |
| Lung cancer | 119 (57.2) | 55 (51.4) | 30 (75.0) | 34 (55.7) |  |
| Liver cancer | 10 (4.8) | 3 (2.8) | 2 (5.0) | 5 (8.2) |  |
| Gastrointestinal cancer | 16 (7.7) | 9 (8.4) | 3 (7.5) | 4 (6.6) |  |
| Breast cancer | 20 (9.6) | 20 (18.7) | 0 (0.0) | 0 (0.0) |  |
| Others | 43 (20.7) | 20 (18.7) | 5 (12.5) | 18 (29.5) |  |
| Visceral metastasis (no/yes, %) | 118/90 (56.7/43.3) | 67/40 (62.6/37.4) | 26/14 (65.0/35.0) | 25/36 (41.0/59.0) | 0.012 |
| Surgery for primary cancer site (%) | |  |  |  | 0.647 |
| Open surgery | 41 (19.7) | 24 (22.4) | 8 (20.0) | 9 (14.8) |  |
| Minimally invasive surgery | 43 (20.7) | 24 (22.4) | 8 (20.0) | 11 (18.0) |  |
| None | 124 (59.6) | 59 (55.1) | 24 (60.0) | 41 (67.2) |  |
| Surgery for spine metastasis (%) | |  |  |  | 0.762 |
| Open surgery | 33 (15.9) | 18 (16.8) | 6 (15.0) | 9 (14.8) |  |
| Minimally invasive surgery | 114 (54.8) | 54 (50.5) | 23 (57.5) | 37 (60.7) |  |
| None | 61 (29.3) | 35 (32.7) | 11 (27.5) | 15 (24.6) |  |
| Radiotherapy (no/yes, %) | 82/126 (39.4/60.6) | 46/61 (43.0/57.0) | 18/22 (45.0/55.0) | 18/43 (29.5/70.5) | 0.165 |
| Chemotherapy (no/yes, %) | 82/126 (39.4/60.6) | 48/59 (44.9/55.1) | 15/25 (37.5/62.5) | 19/42 (31.1/68.9) | 0.209 |
| Economic burden due to cancer treatments (%) | |  |  |  | 0.385 |
| None | 6 (2.9) | 3 (2.8) | 1 (2.5) | 2 (3.3) |  |
| Mild | 22 (10.6) | 13 (12.1) | 7 (17.5) | 2 (3.3) |  |
| Moderate | 67 (32.2) | 35 (32.7) | 13 (32.5) | 19 (31.1) |  |
| Severe | 113 (54.3) | 56 (52.3) | 19 (47.5) | 38 (62.3) |  |
| Having an uncompleted life goal (no/yes, %) | 50/158 (24.0/76.0) | 28/79 (26.2/73.8) | 10/30 (25.0/75.0) | 12/49 (19.7/80.3) | 0.630 |
| ECOG scores (%) |  |  |  |  | <0.001 |
| 0 | 14 (6.7) | 12 (11.2) | 2 (5.0) | 0 (0.0) |  |
| 1 | 71 (34.1) | 54 (50.5) | 15 (37.5) | 2 (3.3) |  |
| 2 | 62 (29.8) | 27 (25.2) | 13 (32.5) | 22 (36.1) |  |
| 3 | 24 (11.5) | 7 (6.5) | 3 (7.5) | 14 (23.0) |  |
| 4 | 37 (17.8) | 7 (6.5) | 7 (17.5) | 23 (37.7) |  |
| Anxiety (%) |  |  |  |  | <0.001 |
| No | 99 (47.6) | 80 (74.8) | 16 (40.0) | 3 (4.9) |  |
| Skeptical | 43 (20.7) | 22 (20.6) | 11 (27.5) | 10 (16.4) |  |
| Yes | 66 (31.7) | 5 (4.7) | 13 (32.5) | 48 (78.7) |  |
| Depression (%) |  |  |  |  | <0.001 |
| No | 107 (51.4) | 107 (100.0) | 0 (0.0) | 0 (0.0) |  |
| Skeptical | 40 (19.2) | 0 (0.0) | 40 (100.0) | 0 (0.0) |  |
| Yes | 61 (29.3) | 0 (0.0) | 0 (0.0) | 61 (100.0) |  |
| Relatively poor quality of life (no/yes, %) | 102/106 (49.0/51.0) | 78/29 (72.9/27.1) | 16/24 (40.0/60.0) | 8/53 (13.1/86.9) | <0.001 |
| FACT-G score (mean (SD)) | 60.32 (20.41) | 71.47 (19.20) | 55.65 (14.32) | 43.84 (11.88) | <0.001 |
| Physical well-being (mean (SD)) | 14.41 (7.22) | 17.75 (6.08) | 13.47 (6.74) | 9.18 (6.07) | <0.001 |
| Social well-being (mean (SD)) | 18.62 (5.82) | 20.20 (5.65) | 16.92 (5.92) | 16.95 (5.34) | <0.001 |
| Emotional well-being (mean (SD)) | 14.24 (5.70) | 16.93 (5.11) | 13.07 (4.78) | 10.28 (4.59) | <0.001 |
| Functional well-being (mean (SD)) | 13.05 (7.14) | 16.59 (6.51) | 12.18 (5.26) | 7.43 (5.28) | <0.001 |
| *Abbreviations: ECOG eastern cooperative oncology group; FACT-G functional assessment of cancer therapy-general; SD standard deviation.* | | | | | |
