# Supplementary material for: A novel nomogram to stratify quality of life among advanced cancer patients with spinal metastatic disease after examining demographics, dietary habits, therapeutic interventions, and mental health status
Source: BMC Cancer. 2022 Nov 23;22:1205. doi: 10.1186/s12885-022-10294-z (PMC9694561; doi:10.1186/s12885-022-10294-z)
Supplement: Supplementary file 6 — Additional file 6. [file 12885_2022_10294_MOESM6_ESM.docx]

| **Additional file 6.**  **Supplementary table 3.** Subgroup analysis of patients stratified by anxiety. | | | | | |
| --- | --- | --- | --- | --- | --- |
| Clinical characteristics | Overall | Anxiety | | | P |
|  |  | No | Skeptical | Yes |  |
| n | 208 | 99 | 43 | 66 |  |
| Age (mean (SD), years) | 58.74 (12.01) | 56.71 (13.26) | 56.86 (12.21) | 63.02 (8.39) | 0.002 |
| Sex (male/female, %) | 107/101 (51.4/48.6) | 45/54 (45.5/54.5) | 24/19 (55.8/44.2) | 38/28 (57.6/42.4) | 0.254 |
| Nationality (han/minorities, %) | 201/7 (96.6/3.4) | 92/7 (92.9/7.1) | 43/0 (100.0/0.0) | 66/0 (100.0/0.0) | 0.019 |
| Marital status (married/single, %) | 194/14 (93.3/6.7) | 93/6 (93.9/6.1) | 37/6 (86.0/14.0) | 64/2 (97.0/3.0) | 0.079 |
| Education (%) |  |  |  |  | 0.009 |
| Primary education | 74 (35.6) | 29 (29.3) | 13 (30.2) | 32 (48.5) |  |
| Senior high school | 73 (35.1) | 31 (31.3) | 18 (41.9) | 24 (36.4) |  |
| University or above | 61 (29.3) | 39 (39.4) | 12 (27.9) | 10 (15.2) |  |
| Caregivers (%) |  |  |  |  | 0.299 |
| Spouse | 135 (64.9) | 65 (65.7) | 28 (65.1) | 42 (63.6) |  |
| Other family members | 39 (18.8) | 14 (14.1) | 12 (27.9) | 13 (19.7) |  |
| Support workers | 10 (4.8) | 7 (7.1) | 0 (0.0) | 3 (4.5) |  |
| No caregivers | 24 (11.5) | 13 (13.1) | 3 (7.0) | 8 (12.1) |  |
| Preference to eat vegetables (no/yes, %) | 28/180 (13.5/86.5) | 13/86 (13.1/86.9) | 8/35 (18.6/81.4) | 7/59 (10.6/89.4) | 0.485 |
| Preference to eat roasted food (no/yes, %) | 188/20 (90.4/9.6) | 90/9 (90.9/9.1) | 41/2 (95.3/4.7) | 57/9 (86.4/13.6) | 0.290 |
| Smoking status (%) |  |  |  |  | 0.156 |
| No | 119 (57.2) | 61 (61.6) | 26 (60.5) | 32 (48.5) |  |
| Quitting smoking | 49 (23.6) | 22 (22.2) | 12 (27.9) | 15 (22.7) |  |
| Current smoking | 40 (19.2) | 16 (16.2) | 5 (11.6) | 19 (28.8) |  |
| Drinking status (%) |  |  |  |  | 0.716 |
| No | 153 (73.6) | 74 (74.7) | 32 (74.4) | 47 (71.2) |  |
| Quitting drinking | 39 (18.8) | 20 (20.2) | 7 (16.3) | 12 (18.2) |  |
| Current drinking | 16 (7.7) | 5 (5.1) | 4 (9.3) | 7 (10.6) |  |
| Hypertension (no/yes, %) | 157/51 (75.5/24.5) | 75/24 (75.8/24.2) | 33/10 (76.7/23.3) | 49/17 (74.2/25.8) | 0.953 |
| Diabetes (no/yes, %) | 188/20 (90.4/9.6) | 91/8 (91.9/8.1) | 35/8 (81.4/18.6) | 62/4 (93.9/6.1) | 0.073 |
| Coronary heart disease (no/yes, %) | 192/16 (92.3/7.7) | 94/5 (94.9/5.1) | 41/2 (95.3/4.7) | 57/9 (86.4/13.6) | 0.09 |
| Time since knowing cancer diagnosis (%) | |  |  |  | 0.043 |
| < 3 months | 37 (17.8) | 15 (15.2) | 9 (20.9) | 13 (19.7) |  |
| ≧3 months and < 6 months | 21 (10.1) | 12 (12.1) | 0 (0.0) | 9 (13.6) |  |
| ≧6 months and < 12 months | 21 (10.1) | 8 (8.1) | 9 (20.9) | 4 (6.1) |  |
| ≧12 months | 129 (62.0) | 64 (64.6) | 25 (58.1) | 40 (60.6) |  |
| Primary cancer type (%) |  |  |  |  | 0.003 |
| Lung cancer | 119 (57.2) | 56 (56.6) | 17 (39.5) | 46 (69.7) |  |
| Liver cancer | 10 (4.8) | 4 (4.0) | 2 (4.7) | 4 (6.1) |  |
| Gastrointestinal cancer | 16 (7.7) | 6 (6.1) | 4 (9.3) | 6 (9.1) |  |
| Breast cancer | 20 (9.6) | 16 (16.2) | 4 (9.3) | 0 (0.0) |  |
| Others | 43 (20.7) | 17 (17.2) | 16 (37.2) | 10 (15.2) |  |
| Visceral metastasis (no/yes, %) | 118/90 (56.7/43.3) | 71/28 (71.7/28.3) | 20/23 (46.5/53.5) | 27/39 (40.9/59.1) | <0.001 |
| Surgery for primary cancer site (%) |  |  |  |  | 0.190 |
| Open surgery | 41 (19.7) | 20 (20.2) | 12 (27.9) | 9 (13.6) |  |
| Minimally invasive surgery | 43 (20.7) | 21 (21.2) | 11 (25.6) | 11 (16.7) |  |
| None | 124 (59.6) | 58 (58.6) | 20 (46.5) | 46 (69.7) |  |
| Surgery for spine metastasis (%) |  |  |  |  | 0.147 |
| Open surgery | 33 (15.9) | 11 (11.1) | 11 (25.6) | 11 (16.7) |  |
| Minimally invasive surgery | 114 (54.8) | 54 (54.5) | 20 (46.5) | 40 (60.6) |  |
| None | 61 (29.3) | 34 (34.3) | 12 (27.9) | 15 (22.7) |  |
| Radiotherapy (no/yes, %) | 82/126 (39.4/60.6) | 45/54 (45.5/54.5) | 20/23 (46.5/53.5) | 17/49 (25.8/74.2) | 0.023 |
| Chemotherapy (no/yes, %) | 82/126 (39.4/60.6) | 45/54 (45.5/54.5) | 17/26 (39.5/60.5) | 20/46 (30.3/69.7) | 0.149 |
| Economic burden due to cancer treatments (%) | |  |  |  | 0.024 |
| None | 6 (2.9) | 3 (3.0) | 1 (2.3) | 2 (3.0) |  |
| Mild | 22 (10.6) | 18 (18.2) | 1 (2.3) | 3 (4.5) |  |
| Moderate | 67 (32.2) | 31 (31.3) | 18 (41.9) | 18 (27.3) |  |
| Severe | 113 (54.3) | 47 (47.5) | 23 (53.5) | 43 (65.2) |  |
| Having an uncompleted life goal (no/yes, %) | 50/158 (24.0/76.0) | 37/62 (37.4/62.6) | 4/39 (9.3/90.7) | 9/57 (13.6/86.4) | <0.001 |
| ECOG scores (%) |  |  |  |  | <0.001 |
| 0 | 14 (6.7) | 12 (12.1) | 2 (4.7) | 0 (0.0) |  |
| 1 | 71 (34.1) | 47 (47.5) | 17 (39.5) | 7 (10.6) |  |
| 2 | 62 (29.8) | 26 (26.3) | 19 (44.2) | 17 (25.8) |  |
| 3 | 24 (11.5) | 10 (10.1) | 2 (4.7) | 12 (18.2) |  |
| 4 | 37 (17.8) | 4 (4.0) | 3 (7.0) | 30 (45.5) |  |
| Anxiety (%) |  |  |  |  | <0.001 |
| No | 99 (47.6) | 99 (100.0) | 0 (0.0) | 0 (0.0) |  |
| Skeptical | 43 (20.7) | 0 (0.0) | 43 (100.0) | 0 (0.0) |  |
| Yes | 66 (31.7) | 0 (0.0) | 0 (0.0) | 66 (100.0) |  |
| Depression (%) |  |  |  |  | <0.001 |
| No | 107 (51.4) | 80 (80.8) | 22 (51.2) | 5 (7.6) |  |
| Skeptical | 40 (19.2) | 16 (16.2) | 11 (25.6) | 13 (19.7) |  |
| Yes | 61 (29.3) | 3 (3.0) | 10 (23.3) | 48 (72.7) |  |
| Relatively poor quality of life (no/yes, %) | 102/106 (49.0/51.0) | 76/23 (76.8/23.2) | 18/25 (41.9/58.1) | 8/58 (12.1/87.9) | <0.001 |
| FACT-G score (mean (SD)) | 60.32 (20.41) | 72.12 (19.28) | 59.30 (13.91) | 43.29 (11.61) | <0.001 |
| Physical well-being (mean (SD)) | 14.41 (7.22) | 18.28 (6.21) | 13.81 (4.60) | 9.00 (6.42) | <0.001 |
| Social well-being (mean (SD)) | 18.62 (5.82) | 19.33 (6.11) | 19.56 (4.59) | 16.92 (5.80) | 0.016 |
| Emotional well-being (mean (SD)) | 14.24 (5.70) | 17.97 (4.15) | 13.44 (4.04) | 9.17 (4.37) | <0.001 |
| Functional well-being (mean (SD)) | 13.05 (7.14) | 16.54 (6.55) | 12.49 (5.72) | 8.20 (5.82) | <0.001 |
| *Abbreviations: ECOG eastern cooperative oncology group; FACT-G functional assessment of cancer therapy-general; SD standard deviation.* | | | | | |
