# Supplementary material for: A novel nomogram to stratify quality of life among advanced cancer patients with spinal metastatic disease after examining demographics, dietary habits, therapeutic interventions, and mental health status
Source: BMC Cancer. 2022 Nov 23;22:1205. doi: 10.1186/s12885-022-10294-z (PMC9694561; doi:10.1186/s12885-022-10294-z)
Supplement: Supplementary file 7 — Additional file 7. [file 12885_2022_10294_MOESM7_ESM.docx]

| **Additional file 7.**  **Supplementary table 4.** Subgroup analysis of patients stratified by ECOG score. | | | | | | | |
| --- | --- | --- | --- | --- | --- | --- | --- |
| Clinical characteristics | Overall | ECOG score | | | | | P |
|  |  | 0 | 1 | 2 | 3 | 4 |  |
| n | 208 | 14 | 71 | 62 | 24 | 37 |  |
| Age (mean (SD), years) | 58.74 (12.01) | 47.43 (13.85) | 55.13 (12.69) | 62.11 (10.45) | 65.21 (8.32) | 60.11 (9.60) | <0.001 |
| Sex (male/female, %) | 107/101 (51.4/48.6) | 8/6 (57.1/42.9) | 25/46 (35.2/64.8) | 44/18 (71.0/29.0) | 13/11 (54.2/45.8) | 17/20 (45.9/54.1) | 0.001 |
| Nationality (han/minorities, %) | 201/7 (96.6/3.4) | 13/1 (92.9/7.1) | 67/4 (94.4/5.6) | 60/2 (96.8/3.2) | 24/0 (100.0/0.0) | 37/0 (100.0/0.0) | 0.424 |
| Marital status (married/single, %) | 194/14 (93.3/6.7) | 14/0 (100.0/0.0) | 64/7 (90.1/9.9) | 58/4 (93.5/6.5) | 21/3 (87.5/12.5) | 37/0 (100.0/0.0) | 0.194 |
| Education (%) |  |  |  |  |  |  | 0.084 |
| Primary education | 74 (35.6) | 4 (28.6) | 16 (22.5) | 26 (41.9) | 10 (41.7) | 18 (48.6) |  |
| Senior high school | 73 (35.1) | 3 (21.4) | 29 (40.8) | 23 (37.1) | 7 (29.2) | 11 (29.7) |  |
| University or above | 61 (29.3) | 7 (50.0) | 26 (36.6) | 13 (21.0) | 7 (29.2) | 8 (21.6) |  |
| Caregivers (%) |  |  |  |  |  |  | 0.060 |
| Spouse | 135 (64.9) | 6 (42.9) | 46 (64.8) | 39 (62.9) | 18 (75.0) | 26 (70.3) |  |
| Other family members | 39 (18.8) | 5 (35.7) | 7 (9.9) | 13 (21.0) | 4 (16.7) | 10 (27.0) |  |
| Support workers | 10 (4.8) | 2 (14.3) | 5 (7.0) | 3 (4.8) | 0 (0.0) | 0 (0.0) |  |
| No caregivers | 24 (11.5) | 1 (7.1) | 13 (18.3) | 7 (11.3) | 2 (8.3) | 1 (2.7) |  |
| Preference to eat vegetables (no/yes, %) | 28/180 (13.5/86.5) | 2/12 (14.3/85.7) | 4/67 (5.6/94.4) | 12/50 (19.4/80.6) | 8/16 (33.3/66.7) | 2/35 (5.4/94.6) | 0.003 |
| Preference to eat roasted food (no/yes, %) | 188/20 (90.4/9.6) | 14/0 (100.0/0.0) | 61/10 (85.9/14.1) | 57/5 (91.9/8.1) | 21/3 (87.5/12.5) | 35/2 (94.6/5.4) | 0.37 |
| Smoking status (%) |  |  |  |  |  |  | 0.718 |
| No | 119 (57.2) | 7 (50.0) | 47 (66.2) | 33 (53.2) | 14 (58.3) | 18 (48.6) |  |
| Quitting smoking | 49 (23.6) | 3 (21.4) | 13 (18.3) | 16 (25.8) | 5 (20.8) | 12 (32.4) |  |
| Current smoking | 40 (19.2) | 4 (28.6) | 11 (15.5) | 13 (21.0) | 5 (20.8) | 7 (18.9) |  |
| Drinking status (%) |  |  |  |  |  |  | 0.334 |
| No | 153 (73.6) | 7 (50.0) | 56 (78.9) | 46 (74.2) | 19 (79.2) | 25 (67.6) |  |
| Quitting drinking | 39 (18.8) | 4 (28.6) | 9 (12.7) | 13 (21.0) | 4 (16.7) | 9 (24.3) |  |
| Current drinking | 16 (7.7) | 3 (21.4) | 6 (8.5) | 3 (4.8) | 1 (4.2) | 3 (8.1) |  |
| Hypertension (no/yes, %) | 157/51 (75.5/24.5) | 13/1 (92.9/7.1) | 55/16 (77.5/22.5) | 48/14 (77.4/22.6) | 11/13 (45.8/54.2) | 30/7 (81.1/18.9) | 0.006 |
| Diabetes (no/yes, %) | 188/20 (90.4/9.6) | 14/0 (100.0/0.0) | 66/5 (93.0/7.0) | 56/6 (90.3/9.7) | 20/4 (83.3/16.7) | 32/5 (86.5/13.5) | 0.399 |
| Coronary heart disease (no/yes, %) | 192/16 (92.3/7.7) | 14/0 (100.0/0.0) | 66/5 (93.0/7.0) | 57/5 (91.9/8.1) | 19/5 (79.2/20.8) | 36/1 (97.3/2.7) | 0.079 |
| Time since knowing cancer diagnosis (%) | |  |  |  |  |  | 0.124 |
| < 3 months | 37 (17.8) | 3 (21.4) | 15 (21.1) | 7 (11.3) | 4 (16.7) | 8 (21.6) |  |
| ≧3 months and < 6 months | 21 (10.1) | 1 (7.1) | 7 (9.9) | 10 (16.1) | 0 (0.0) | 3 (8.1) |  |
| ≧6 months and < 12 months | 21 (10.1) | 4 (28.6) | 4 (5.6) | 9 (14.5) | 1 (4.2) | 3 (8.1) |  |
| ≧12 months | 129 (62.0) | 6 (42.9) | 45 (63.4) | 36 (58.1) | 19 (79.2) | 23 (62.2) |  |
| Primary cancer type (%) |  |  |  |  |  |  | 0.001 |
| Lung cancer | 119 (57.2) | 6 (42.9) | 38 (53.5) | 38 (61.3) | 10 (41.7) | 27 (73.0) |  |
| Liver cancer | 10 (4.8) | 2 (14.3) | 1 (1.4) | 2 (3.2) | 5 (20.8) | 0 (0.0) |  |
| Gastrointestinal cancer | 16 (7.7) | 2 (14.3) | 4 (5.6) | 4 (6.5) | 1 (4.2) | 5 (13.5) |  |
| Breast cancer | 20 (9.6) | 0 (0.0) | 13 (18.3) | 3 (4.8) | 4 (16.7) | 0 (0.0) |  |
| Others | 43 (20.7) | 4 (28.6) | 15 (21.1) | 15 (24.2) | 4 (16.7) | 5 (13.5) |  |
| Visceral metastasis (no/yes, %) | 118/90 (56.7/43.3) | 12/2 (85.7/14.3) | 57/14 (80.3/19.7) | 28/34 (45.2/54.8) | 12/12 (50.0/50.0) | 9/28 (24.3/75.7) | <0.001 |
| Surgery for primary cancer site (%) |  |  |  |  |  |  | 0.144 |
| Open surgery | 41 (19.7) | 2 (14.3) | 14 (19.7) | 17 (27.4) | 1 (4.2) | 7 (18.9) |  |
| Minimally invasive surgery | 43 (20.7) | 6 (42.9) | 17 (23.9) | 9 (14.5) | 5 (20.8) | 6 (16.2) |  |
| None | 124 (59.6) | 6 (42.9) | 40 (56.3) | 36 (58.1) | 18 (75.0) | 24 (64.9) |  |
| Surgery for spine metastasis (%) |  |  |  |  |  |  | 0.032 |
| Open surgery | 33 (15.9) | 0 (0.0) | 8 (11.3) | 12 (19.4) | 2 (8.3) | 11 (29.7) |  |
| Minimally invasive surgery | 114 (54.8) | 8 (57.1) | 35 (49.3) | 37 (59.7) | 14 (58.3) | 20 (54.1) |  |
| None | 61 (29.3) | 6 (42.9) | 28 (39.4) | 13 (21.0) | 8 (33.3) | 6 (16.2) |  |
| Radiotherapy (no/yes, %) | 82/126 (39.4/60.6) | 8/6 (57.1/42.9) | 39/32 (54.9/45.1) | 20/42 (32.3/67.7) | 7/17 (29.2/70.8) | 8/29 (21.6/78.4) | 0.003 |
| Chemotherapy (no/yes, %) | 82/126 (39.4/60.6) | 10/4 (71.4/28.6) | 34/37 (47.9/52.1) | 24/38 (38.7/61.3) | 5/19 (20.8/79.2) | 9/28 (24.3/75.7) | 0.004 |
| Economic burden due to cancer treatments (%) | |  |  |  |  |  | 0.811 |
| None | 6 (2.9) | 1 (7.1) | 2 (2.8) | 1 (1.6) | 0 (0.0) | 2 (5.4) |  |
| Mild | 22 (10.6) | 2 (14.3) | 9 (12.7) | 7 (11.3) | 3 (12.5) | 1 (2.7) |  |
| Moderate | 67 (32.2) | 4 (28.6) | 24 (33.8) | 18 (29.0) | 10 (41.7) | 11 (29.7) |  |
| Severe | 113 (54.3) | 7 (50.0) | 36 (50.7) | 36 (58.1) | 11 (45.8) | 23 (62.2) |  |
| Having an uncompleted life goal (no/yes, %) | 50/158 (24.0/76.0) | 7/7 (50.0/50.0) | 16/55 (22.5/77.5) | 14/48 (22.6/77.4) | 3/21 (12.5/87.5) | 10/27 (27.0/73.0) | 0.123 |
| ECOG scores (%) |  |  |  |  |  |  | <0.001 |
| 0 | 14 (6.7) | 14 (100.0) | 0 (0.0) | 0 (0.0) | 0 (0.0) | 0 (0.0) |  |
| 1 | 71 (34.1) | 0 (0.0) | 71 (100.0) | 0 (0.0) | 0 (0.0) | 0 (0.0) |  |
| 2 | 62 (29.8) | 0 (0.0) | 0 (0.0) | 62 (100.0) | 0 (0.0) | 0 (0.0) |  |
| 3 | 24 (11.5) | 0 (0.0) | 0 (0.0) | 0 (0.0) | 24 (100.0) | 0 (0.0) |  |
| 4 | 37 (17.8) | 0 (0.0) | 0 (0.0) | 0 (0.0) | 0 (0.0) | 37 (100.0) |  |
| Anxiety (%) |  |  |  |  |  |  | <0.001 |
| No | 99 (47.6) | 12 (85.7) | 47 (66.2) | 26 (41.9) | 10 (41.7) | 4 (10.8) |  |
| Skeptical | 43 (20.7) | 2 (14.3) | 17 (23.9) | 19 (30.6) | 2 (8.3) | 3 (8.1) |  |
| Yes | 66 (31.7) | 0 (0.0) | 7 (9.9) | 17 (27.4) | 12 (50.0) | 30 (81.1) |  |
| Depression (%) |  |  |  |  |  |  | <0.001 |
| No | 107 (51.4) | 12 (85.7) | 54 (76.1) | 27 (43.5) | 7 (29.2) | 7 (18.9) |  |
| Skeptical | 40 (19.2) | 2 (14.3) | 15 (21.1) | 13 (21.0) | 3 (12.5) | 7 (18.9) |  |
| Yes | 61 (29.3) | 0 (0.0) | 2 (2.8) | 22 (35.5) | 14 (58.3) | 23 (62.2) |  |
| Relatively poor quality of life (no/yes, %) | 102/106 (49.0/51.0) | 12/2 (85.7/14.3) | 54/17 (76.1/23.9) | 24/38 (38.7/61.3) | 6/18 (25.0/75.0) | 6/31 (16.2/83.8) | <0.001 |
| FACT-G score (mean (SD)) | 60.32 (20.41) | 82.36 (26.07) | 73.01 (17.58) | 56.21 (12.26) | 45.96 (15.62) | 43.84 (13.67) | <0.001 |
| Physical well-being (mean (SD)) | 14.41 (7.22) | 20.00 (7.17) | 19.79 (4.40) | 14.02 (4.10) | 9.04 (5.45) | 6.14 (6.09) | <0.001 |
| Social well-being (mean (SD)) | 18.62 (5.82) | 22.14 (7.35) | 19.69 (5.71) | 17.39 (4.82) | 15.21 (5.52) | 19.49 (5.89) | <0.001 |
| Emotional well-being (mean (SD)) | 14.24 (5.70) | 19.43 (6.52) | 16.39 (4.54) | 14.34 (4.81) | 11.79 (5.91) | 9.57 (4.86) | <0.001 |
| Functional well-being (mean (SD)) | 13.05 (7.14) | 20.79 (6.80) | 17.14 (6.39) | 10.47 (4.60) | 9.92 (4.37) | 8.65 (7.38) | <0.001 |
| *Abbreviations: ECOG eastern cooperative oncology group; FACT-G functional assessment of cancer therapy-general; SD standard deviation.* | | | | | | | |
