# Supplementary material for: A novel nomogram to stratify quality of life among advanced cancer patients with spinal metastatic disease after examining demographics, dietary habits, therapeutic interventions, and mental health status
Source: BMC Cancer. 2022 Nov 23;22:1205. doi: 10.1186/s12885-022-10294-z (PMC9694561; doi:10.1186/s12885-022-10294-z)
Supplement: Supplementary file 8 — Additional file 8. [file 12885_2022_10294_MOESM8_ESM.docx]

| **Additional file 8.**  **Supplementary table 5.** Subgroup analysis of patients stratified by having an uncompleted life goal. | | | | |
| --- | --- | --- | --- | --- |
| Clinical characteristics | Overall | Having an uncompleted life goal | | P |
|  |  | 0 | 1 |  |
| n | 208 | 50 | 158 |  |
| Age (mean (SD), years) | 58.74 (12.01) | 60.36 (12.49) | 58.23 (11.84) | 0.275 |
| Sex (male/female, %) | 107/101 (51.4/48.6) | 22/28 (44.0/56.0) | 85/73 (53.8/46.2) | 0.296 |
| Nationality (han/minorities, %) | 201/7 (96.6/3.4) | 49/1 (98.0/2.0) | 152/6 (96.2/3.8) | 0.869 |
| Marital status (married/single, %) | 194/14 (93.3/6.7) | 49/1 (98.0/2.0) | 145/13 (91.8/8.2) | 0.227 |
| Education (%) |  |  |  | 0.015 |
| Primary education | 74 (35.6) | 26 (52.0) | 48 (30.4) |  |
| Senior high school | 73 (35.1) | 11 (22.0) | 62 (39.2) |  |
| University or above | 61 (29.3) | 13 (26.0) | 48 (30.4) |  |
| Caregivers (%) |  |  |  | 0.365 |
| Spouse | 135 (64.9) | 28 (56.0) | 107 (67.7) |  |
| Other family members | 39 (18.8) | 12 (24.0) | 27 (17.1) |  |
| Support workers | 10 (4.8) | 4 (8.0) | 6 (3.8) |  |
| No caregivers | 24 (11.5) | 6 (12.0) | 18 (11.4) |  |
| Preference to eat vegetables (no/yes, %) | 28/180 (13.5/86.5) | 6/44 (12.0/88.0) | 22/136 (13.9/86.1) | 0.913 |
| Preference to eat roasted food (no/yes, %) | 188/20 (90.4/9.6) | 50/0 (100.0/0.0) | 138/20 (87.3/12.7) | 0.018 |
| Smoking status (%) |  |  |  | 0.793 |
| No | 119 (57.2) | 30 (60.0) | 89 (56.3) |  |
| Quitting smoking | 49 (23.6) | 10 (20.0) | 39 (24.7) |  |
| Current smoking | 40 (19.2) | 10 (20.0) | 30 (19.0) |  |
| Drinking status (%) |  |  |  | 0.372 |
| No | 153 (73.6) | 40 (80.0) | 113 (71.5) |  |
| Quitting drinking | 39 (18.8) | 6 (12.0) | 33 (20.9) |  |
| Current drinking | 16 (7.7) | 4 (8.0) | 12 (7.6) |  |
| Hypertension (no/yes, %) | 157/51 (75.5/24.5) | 35/15 (70.0/30.0) | 122/36 (77.2/22.8) | 0.398 |
| Diabetes (no/yes, %) | 188/20 (90.4/9.6) | 45/5 (90.0/10.0) | 143/15 (90.5/9.5) | 1.000 |
| Coronary heart disease (no/yes, %) | 192/16 (92.3/7.7) | 47/3 (94.0/6.0) | 145/13 (91.8/8.2) | 0.833 |
| Time since knowing cancer diagnosis (%) | |  |  | 0.604 |
| < 3 months | 37 (17.8) | 7 (14.0) | 30 (19.0) |  |
| ≧3 months and < 6 months | 21 (10.1) | 7 (14.0) | 14 (8.9) |  |
| ≧6 months and < 12 months | 21 (10.1) | 4 (8.0) | 17 (10.8) |  |
| ≧12 months | 129 (62.0) | 32 (64.0) | 97 (61.4) |  |
| Primary cancer type (%) |  |  |  | 0.702 |
| Lung cancer | 119 (57.2) | 28 (56.0) | 91 (57.6) |  |
| Liver cancer | 10 (4.8) | 1 (2.0) | 9 (5.7) |  |
| Gastrointestinal cancer | 16 (7.7) | 4 (8.0) | 12 (7.6) |  |
| Breast cancer | 20 (9.6) | 4 (8.0) | 16 (10.1) |  |
| Others | 43 (20.7) | 13 (26.0) | 30 (19.0) |  |
| Visceral metastasis (no/yes, %) | 118/90 (56.7/43.3) | 34/16 (68.0/32.0) | 84/74 (53.2/46.8) | 0.093 |
| Surgery for primary cancer site (%) | |  |  | 0.340 |
| Open surgery | 41 (19.7) | 9 (18.0) | 32 (20.3) |  |
| Minimally invasive surgery | 43 (20.7) | 14 (28.0) | 29 (18.4) |  |
| None | 124 (59.6) | 27 (54.0) | 97 (61.4) |  |
| Surgery for spine metastasis (%) |  |  |  | 0.201 |
| Open surgery | 33 (15.9) | 5 (10.0) | 28 (17.7) |  |
| Minimally invasive surgery | 114 (54.8) | 26 (52.0) | 88 (55.7) |  |
| None | 61 (29.3) | 19 (38.0) | 42 (26.6) |  |
| Radiotherapy (no/yes, %) | 82/126 (39.4/60.6) | 22/28 (44.0/56.0) | 60/98 (38.0/62.0) | 0.553 |
| Chemotherapy (no/yes, %) | 82/126 (39.4/60.6) | 28/22 (56.0/44.0) | 54/104 (34.2/65.8) | 0.010 |
| Economic burden due to cancer treatments (%) | |  |  | <0.001 |
| None | 6 (2.9) | 5 (10.0) | 1 (0.6) |  |
| Mild | 22 (10.6) | 12 (24.0) | 10 (6.3) |  |
| Moderate | 67 (32.2) | 16 (32.0) | 51 (32.3) |  |
| Severe | 113 (54.3) | 17 (34.0) | 96 (60.8) |  |
| Having an uncompleted life goal (no/yes, %) | 50/158 (24.0/76.0) | 50/0 (100.0/0.0) | 0/158 (0.0/100.0) | <0.001 |
| ECOG scores (%) |  |  |  | 0.123 |
| 0 | 14 (6.7) | 7 (14.0) | 7 (4.4) |  |
| 1 | 71 (34.1) | 16 (32.0) | 55 (34.8) |  |
| 2 | 62 (29.8) | 14 (28.0) | 48 (30.4) |  |
| 3 | 24 (11.5) | 3 (6.0) | 21 (13.3) |  |
| 4 | 37 (17.8) | 10 (20.0) | 27 (17.1) |  |
| Anxiety (%) |  |  |  | <0.001 |
| No | 99 (47.6) | 37 (74.0) | 62 (39.2) |  |
| Skeptical | 43 (20.7) | 4 (8.0) | 39 (24.7) |  |
| Yes | 66 (31.7) | 9 (18.0) | 57 (36.1) |  |
| Depression (%) |  |  |  | 0.630 |
| No | 107 (51.4) | 28 (56.0) | 79 (50.0) |  |
| Skeptical | 40 (19.2) | 10 (20.0) | 30 (19.0) |  |
| Yes | 61 (29.3) | 12 (24.0) | 49 (31.0) |  |
| Relatively poor quality of life (no/yes, %) | 102/106 (49.0/51.0) | 36/14 (72.0/28.0) | 66/92 (41.8/58.2) | <0.001 |
| FACT-G score (mean (SD)) | 60.32 (20.41) | 71.12 (22.37) | 56.91 (18.56) | <0.001 |
| Physical well-being (mean (SD)) | 14.41 (7.22) | 16.78 (7.72) | 13.66 (6.91) | 0.008 |
| Social well-being (mean (SD)) | 18.62 (5.82) | 20.18 (5.85) | 18.12 (5.74) | 0.029 |
| Emotional well-being (mean (SD)) | 14.24 (5.70) | 17.82 (5.36) | 13.11 (5.34) | <0.001 |
| Functional well-being (mean (SD)) | 13.05 (7.14) | 16.34 (8.34) | 12.01 (6.40) | <0.001 |
| *Abbreviations: ECOG eastern cooperative oncology group; FACT-G functional assessment of cancer therapy-general; SD standard deviation.* | | | | |
