# Supplementary material for: A novel nomogram to stratify quality of life among advanced cancer patients with spinal metastatic disease after examining demographics, dietary habits, therapeutic interventions, and mental health status
Source: BMC Cancer. 2022 Nov 23;22:1205. doi: 10.1186/s12885-022-10294-z (PMC9694561; doi:10.1186/s12885-022-10294-z)
Supplement: Supplementary file 9 — Additional file 9. [file 12885_2022_10294_MOESM9_ESM.docx]

| **Additional file 9.**  **Supplementary table 6.** Subgroup analysis of patients stratified by preference for eating vegetables. | | | | |
| --- | --- | --- | --- | --- |
| Clinical characteristics | Overall | Preference for eating vegetables | | P |
|  |  | No | Yes |  |
| n | 208 | 28 | 180 |  |
| Age (mean (SD), years) | 58.74 (12.01) | 58.75 (12.86) | 58.74 (11.91) | 0.996 |
| Sex (male/female, %) | 107/101 (51.4/48.6) | 20/8 (71.4/28.6) | 87/93 (48.3/51.7) | 0.038 |
| Nationality (han/minorities, %) | 201/7 (96.6/3.4) | 28/0 (100.0/0.0) | 173/7 (96.1/3.9) | 0.618 |
| Marital status (married/single, %) | 194/14 (93.3/6.7) | 23/5 (82.1/17.9) | 171/9 (95.0/5.0) | 0.034 |
| Education (%) |  |  |  | 0.638 |
| Primary education | 74 (35.6) | 8 (28.6) | 66 (36.7) |  |
| Senior high school | 73 (35.1) | 10 (35.7) | 63 (35.0) |  |
| University or above | 61 (29.3) | 10 (35.7) | 51 (28.3) |  |
| Caregivers (%) |  |  |  | 0.820 |
| Spouse | 135 (64.9) | 18 (64.3) | 117 (65.0) |  |
| Other family members | 39 (18.8) | 4 (14.3) | 35 (19.4) |  |
| Support workers | 10 (4.8) | 2 (7.1) | 8 (4.4) |  |
| No caregivers | 24 (11.5) | 4 (14.3) | 20 (11.1) |  |
| Preference to eat vegetables (no/yes, %) | 28/180 (13.5/86.5) | 28/0 (100.0/0.0) | 0/180 (0.0/100.0) | <0.001 |
| Preference to eat roasted food (no/yes, %) | 188/20 (90.4/9.6) | 23/5 (82.1/17.9) | 165/15 (91.7/8.3) | 0.213 |
| Smoking status (%) |  |  |  | 0.015 |
| No | 119 (57.2) | 12 (42.9) | 107 (59.4) |  |
| Quitting smoking | 49 (23.6) | 5 (17.9) | 44 (24.4) |  |
| Current smoking | 40 (19.2) | 11 (39.3) | 29 (16.1) |  |
| Drinking status (%) |  |  |  | 0.045 |
| No | 153 (73.6) | 16 (57.1) | 137 (76.1) |  |
| Quitting drinking | 39 (18.8) | 7 (25.0) | 32 (17.8) |  |
| Current drinking | 16 (7.7) | 5 (17.9) | 11 (6.1) |  |
| Hypertension (no/yes, %) | 157/51 (75.5/24.5) | 19/9 (67.9/32.1) | 138/42 (76.7/23.3) | 0.440 |
| Diabetes (no/yes, %) | 188/20 (90.4/9.6) | 24/4 (85.7/14.3) | 164/16 (91.1/8.9) | 0.578 |
| Coronary heart disease (no/yes, %) | 192/16 (92.3/7.7) | 25/3 (89.3/10.7) | 167/13 (92.8/7.2) | 0.792 |
| Time since knowing cancer diagnosis (%) | |  |  | 0.035 |
| < 3 months | 37 (17.8) | 10 (35.7) | 27 (15.0) |  |
| ≧3 months and < 6 months | 21 (10.1) | 2 (7.1) | 19 (10.6) |  |
| ≧6 months and < 12 months | 21 (10.1) | 4 (14.3) | 17 (9.4) |  |
| ≧12 months | 129 (62.0) | 12 (42.9) | 117 (65.0) |  |
| Primary cancer type (%) |  |  |  | 0.061 |
| Lung cancer | 119 (57.2) | 17 (60.7) | 102 (56.7) |  |
| Liver cancer | 10 (4.8) | 3 (10.7) | 7 (3.9) |  |
| Gastrointestinal cancer | 16 (7.7) | 0 (0.0) | 16 (8.9) |  |
| Breast cancer | 20 (9.6) | 0 (0.0) | 20 (11.1) |  |
| Others | 43 (20.7) | 8 (28.6) | 35 (19.4) |  |
| Visceral metastasis (no/yes, %) | 118/90 (56.7/43.3) | 15/13 (53.6/46.4) | 103/77 (57.2/42.8) | 0.875 |
| Surgery for primary cancer site (%) | |  |  | 0.001 |
| Open surgery | 41 (19.7) | 1 (3.6) | 40 (22.2) |  |
| Minimally invasive surgery | 43 (20.7) | 13 (46.4) | 30 (16.7) |  |
| None | 124 (59.6) | 14 (50.0) | 110 (61.1) |  |
| Surgery for spine metastasis (%) | |  |  | 0.686 |
| Open surgery | 33 (15.9) | 3 (10.7) | 30 (16.7) |  |
| Minimally invasive surgery | 114 (54.8) | 17 (60.7) | 97 (53.9) |  |
| None | 61 (29.3) | 8 (28.6) | 53 (29.4) |  |
| Radiotherapy (no/yes, %) | 82/126 (39.4/60.6) | 13/15 (46.4/53.6) | 69/111 (38.3/61.7) | 0.543 |
| Chemotherapy (no/yes, %) | 82/126 (39.4/60.6) | 6/22 (21.4/78.6) | 76/104 (42.2/57.8) | 0.059 |
| Economic burden due to cancer treatments (%) | |  |  | 0.526 |
| None | 6 (2.9) | 0 (0.0) | 6 (3.3) |  |
| Mild | 22 (10.6) | 4 (14.3) | 18 (10.0) |  |
| Moderate | 67 (32.2) | 11 (39.3) | 56 (31.1) |  |
| Severe | 113 (54.3) | 13 (46.4) | 100 (55.6) |  |
| Having an uncompleted life goal (no/yes, %) | 50/158 (24.0/76.0) | 6/22 (21.4/78.6) | 44/136 (24.4/75.6) | 0.913 |
| ECOG scores (%) |  |  |  | 0.003 |
| 0 | 14 (6.7) | 2 (7.1) | 12 (6.7) |  |
| 1 | 71 (34.1) | 4 (14.3) | 67 (37.2) |  |
| 2 | 62 (29.8) | 12 (42.9) | 50 (27.8) |  |
| 3 | 24 (11.5) | 8 (28.6) | 16 (8.9) |  |
| 4 | 37 (17.8) | 2 (7.1) | 35 (19.4) |  |
| Anxiety (%) |  |  |  | 0.485 |
| No | 99 (47.6) | 13 (46.4) | 86 (47.8) |  |
| Skeptical | 43 (20.7) | 8 (28.6) | 35 (19.4) |  |
| Yes | 66 (31.7) | 7 (25.0) | 59 (32.8) |  |
| Depression (%) |  |  |  | <0.001 |
| No | 107 (51.4) | 7 (25.0) | 100 (55.6) |  |
| Skeptical | 40 (19.2) | 13 (46.4) | 27 (15.0) |  |
| Yes | 61 (29.3) | 8 (28.6) | 53 (29.4) |  |
| Relatively poor quality of life (no/yes, %) | 102/106 (49.0/51.0) | 8/20 (28.6/71.4) | 94/86 (52.2/47.8) | 0.034 |
| FACT-G score (mean (SD)) | 60.32 (20.41) | 46.00 (16.43) | 62.55 (20.10) | <0.001 |
| Physical well-being (mean (SD)) | 14.41 (7.22) | 10.96 (6.09) | 14.95 (7.25) | 0.006 |
| Social well-being (mean (SD)) | 18.62 (5.82) | 13.61 (5.80) | 19.39 (5.44) | <0.001 |
| Emotional well-being (mean (SD)) | 14.24 (5.70) | 11.61 (5.51) | 14.65 (5.63) | 0.008 |
| Functional well-being (mean (SD)) | 13.05 (7.14) | 9.82 (4.57) | 13.56 (7.34) | 0.010 |
| *Abbreviations: ECOG eastern cooperative oncology group; FACT-G functional assessment of cancer therapy-general; SD standard deviation.* | | | | |
