# Supplementary material for: A novel nomogram to stratify quality of life among advanced cancer patients with spinal metastatic disease after examining demographics, dietary habits, therapeutic interventions, and mental health status
Source: BMC Cancer. 2022 Nov 23;22:1205. doi: 10.1186/s12885-022-10294-z (PMC9694561; doi:10.1186/s12885-022-10294-z)
Supplement: Supplementary file 10 — Additional file 10. [file 12885_2022_10294_MOESM10_ESM.docx]

| **Additional file 10.**  **Supplementary table 7.** Subgroup analysis of patients stratified by visceral metastases. | | | | |
| --- | --- | --- | --- | --- |
| Clinical characteristics | Overall | Visceral metastases | | P |
|  |  | No | Yes |  |
| n | 208 | 118 | 90 |  |
| Age (mean (SD), years) | 58.74 (12.01) | 58.18 (12.59) | 59.48 (11.22) | 0.441 |
| Sex (male/female, %) | 107/101 (51.4/48.6) | 53/65 (44.9/55.1) | 54/36 (60.0/40.0) | 0.044 |
| Nationality (han/minorities, %) | 201/7 (96.6/3.4) | 113/5 (95.8/4.2) | 88/2 (97.8/2.2) | 0.682 |
| Marital status (married/single, %) | 194/14 (93.3/6.7) | 110/8 (93.2/6.8) | 84/6 (93.3/6.7) | 1.000 |
| Education (%) |  |  |  | 0.054 |
| Primary education | 74 (35.6) | 34 (28.8) | 40 (44.4) |  |
| Senior high school | 73 (35.1) | 44 (37.3) | 29 (32.2) |  |
| University or above | 61 (29.3) | 40 (33.9) | 21 (23.3) |  |
| Caregivers (%) |  |  |  | 0.004 |
| Spouse | 135 (64.9) | 68 (57.6) | 67 (74.4) |  |
| Other family members | 39 (18.8) | 22 (18.6) | 17 (18.9) |  |
| Support workers | 10 (4.8) | 10 (8.5) | 0 (0.0) |  |
| No caregivers | 24 (11.5) | 18 (15.3) | 6 (6.7) |  |
| Preference to eat vegetables (no/yes, %) | 28/180 (13.5/86.5) | 15/103 (12.7/87.3) | 13/77 (14.4/85.6) | 0.875 |
| Preference to eat roasted food (no/yes, %) | 188/20 (90.4/9.6) | 106/12 (89.8/10.2) | 82/8 (91.1/8.9) | 0.942 |
| Smoking status (%) |  |  |  | 0.080 |
| No | 119 (57.2) | 73 (61.9) | 46 (51.1) |  |
| Quitting smoking | 49 (23.6) | 21 (17.8) | 28 (31.1) |  |
| Current smoking | 40 (19.2) | 24 (20.3) | 16 (17.8) |  |
| Drinking status (%) |  |  |  | 0.084 |
| No | 153 (73.6) | 86 (72.9) | 67 (74.4) |  |
| Quitting drinking | 39 (18.8) | 19 (16.1) | 20 (22.2) |  |
| Current drinking | 16 (7.7) | 13 (11.0) | 3 (3.3) |  |
| Hypertension (no/yes, %) | 157/51 (75.5/24.5) | 88/30 (74.6/25.4) | 69/21 (76.7/23.3) | 0.854 |
| Diabetes (no/yes, %) | 188/20 (90.4/9.6) | 109/9 (92.4/7.6) | 79/11 (87.8/12.2) | 0.381 |
| Coronary heart disease (no/yes, %) | 192/16 (92.3/7.7) | 107/11 (90.7/9.3) | 85/5 (94.4/5.6) | 0.455 |
| Time since knowing cancer diagnosis (%) | |  |  | 0.237 |
| < 3 months | 37 (17.8) | 24 (20.3) | 13 (14.4) |  |
| ≧3 months and < 6 months | 21 (10.1) | 11 (9.3) | 10 (11.1) |  |
| ≧6 months and < 12 months | 21 (10.1) | 8 (6.8) | 13 (14.4) |  |
| ≧12 months | 129 (62.0) | 75 (63.6) | 54 (60.0) |  |
| Primary cancer type (%) |  |  |  | 0.353 |
| Lung cancer | 119 (57.2) | 66 (55.9) | 53 (58.9) |  |
| Liver cancer | 10 (4.8) | 4 (3.4) | 6 (6.7) |  |
| Gastrointestinal cancer | 16 (7.7) | 7 (5.9) | 9 (10.0) |  |
| Breast cancer | 20 (9.6) | 12 (10.2) | 8 (8.9) |  |
| Others | 43 (20.7) | 29 (24.6) | 14 (15.6) |  |
| Visceral metastasis (no/yes, %) | 118/90 (56.7/43.3) | 118/0 (100.0/0.0) | 0/90 (0.0/100.0) | <0.001 |
| Surgery for primary cancer site (%) |  |  |  | 0.009 |
| Open surgery | 41 (19.7) | 17 (14.4) | 24 (26.7) |  |
| Minimally invasive surgery | 43 (20.7) | 32 (27.1) | 11 (12.2) |  |
| None | 124 (59.6) | 69 (58.5) | 55 (61.1) |  |
| Surgery for spine metastasis (%) |  |  |  | 0.089 |
| Open surgery | 33 (15.9) | 13 (11.0) | 20 (22.2) |  |
| Minimally invasive surgery | 114 (54.8) | 69 (58.5) | 45 (50.0) |  |
| None | 61 (29.3) | 36 (30.5) | 25 (27.8) |  |
| Radiotherapy (no/yes, %) | 82/126 (39.4/60.6) | 62/56 (52.5/47.5) | 20/70 (22.2/77.8) | <0.001 |
| Chemotherapy (no/yes, %) | 82/126 (39.4/60.6) | 60/58 (50.8/49.2) | 22/68 (24.4/75.6) | <0.001 |
| Economic burden due to cancer treatments (%) | |  |  | 0.082 |
| None | 6 (2.9) | 3 (2.5) | 3 (3.3) |  |
| Mild | 22 (10.6) | 18 (15.3) | 4 (4.4) |  |
| Moderate | 67 (32.2) | 38 (32.2) | 29 (32.2) |  |
| Severe | 113 (54.3) | 59 (50.0) | 54 (60.0) |  |
| Having an uncompleted life goal (no/yes, %) | 50/158 (24.0/76.0) | 34/84 (28.8/71.2) | 16/74 (17.8/82.2) | 0.093 |
| ECOG scores (%) |  |  |  | <0.001 |
| 0 | 14 (6.7) | 12 (10.2) | 2 (2.2) |  |
| 1 | 71 (34.1) | 57 (48.3) | 14 (15.6) |  |
| 2 | 62 (29.8) | 28 (23.7) | 34 (37.8) |  |
| 3 | 24 (11.5) | 12 (10.2) | 12 (13.3) |  |
| 4 | 37 (17.8) | 9 (7.6) | 28 (31.1) |  |
| Anxiety (%) |  |  |  | <0.001 |
| No | 99 (47.6) | 71 (60.2) | 28 (31.1) |  |
| Skeptical | 43 (20.7) | 20 (16.9) | 23 (25.6) |  |
| Yes | 66 (31.7) | 27 (22.9) | 39 (43.3) |  |
| Depression (%) |  |  |  | 0.012 |
| No | 107 (51.4) | 67 (56.8) | 40 (44.4) |  |
| Skeptical | 40 (19.2) | 26 (22.0) | 14 (15.6) |  |
| Yes | 61 (29.3) | 25 (21.2) | 36 (40.0) |  |
| Relatively poor quality of life (no/yes, %) | 102/106 (49.0/51.0) | 68/50 (57.6/42.4) | 34/56 (37.8/62.2) | 0.007 |
| FACT-G score (mean (SD)) | 60.32 (20.41) | 64.63 (20.93) | 54.68 (18.35) | <0.001 |
| Physical well-being (mean (SD)) | 14.41 (7.22) | 16.54 (6.75) | 11.62 (6.89) | <0.001 |
| Social well-being (mean (SD)) | 18.62 (5.82) | 18.01 (6.49) | 19.41 (4.71) | 0.085 |
| Emotional well-being (mean (SD)) | 14.24 (5.70) | 15.49 (5.22) | 12.60 (5.91) | <0.001 |
| Functional well-being (mean (SD)) | 13.05 (7.14) | 14.58 (7.27) | 11.04 (6.47) | <0.001 |
| *Abbreviations: ECOG eastern cooperative oncology group; FACT-G functional assessment of cancer therapy-general; SD standard deviation.* | | | | |
